# Characterization of diverse populations of sinoatrial node cells and their proliferation potential at single nucleus resolution

**DOI:** 10.1016/j.heliyon.2022.e12708

**Published:** 2022-12-29

**Authors:** Jia-Hua Qu, Richard Telljohann, Rostislav Byshkov, Edward G. Lakatta

**Affiliations:** Laboratory of Cardiovascular Science, Intramural Research Program, National Institute on Aging, National Institutes of Health, Baltimore, MD 21224, USA

**Keywords:** Sinoatrial node, Single nucleus RNA-seq, Pacemaker, Proliferation, Metabolism, Immune

## Abstract

**Background:**

Each heartbeat is initiated in the sinoatrial node (SAN), and although a recent study (GSE130710) using single nucleus RNA-seq had discovered different populations of cell types within SAN tissue, the distinct potential functions of these cell types have not been delineated.

**Methods:**

To infer some special potential functions of different SAN cell clusters, we applied principal component analysis (PCA), t-distributed stochastic neighbor embedding (t-SNE) and uniform manifold approximation and projection (UMAP) to the GSE130710 dataset to reduce dimensions, followed by Pseudotime trajectory and AUCell analyses, ANOVA and Hurdle statistical models, and Gene Ontology (GO) and Kyoto Encyclopedia of Genes and Genomes (KEGG) enrichments to determine functional potential of cell types. Nuclear EdU immuno-labeling of SAN tissue confirmed cell type proliferation.

**Findings:**

We identified elements of a coupled clock system known to drive SAN cell pacemaking within the GSE130710 sinus node myocyte cluster, which, surprisingly, manifested signals of suppressed fatty acid and nitrogen metabolism and reduced immune gene expression. Proliferation signaling was enriched in endocardial, epicardial, epithelial cells, and macrophages, in which, fatty acid and nitrogen metabolic signals were also suppressed, but immune signaling was enhanced. EdU labeling was rare in pacemaker cells but was robust in interstitial cells.

**Interpretation:**

Pacemaker cells that initiate each heartbeat manifest suppressed fatty acid and nitrogen metabolism and limited immune signaling and proliferation potential. In contrast, other populations of SAN cells not directly involved in the initiation of heartbeats, manifest robust proliferation and immune potential, likely to ensure an environment required to sustain healthy SAN tissue pacemaker function.

**Research in context**

Evidence before this study

The heart is composed of different compartments with specialized functions. The sinoatrial node (SAN) which initiates the heartbeat and maintains its normal beating rhythm, harbors many different cell types, including pacemaker cells, mainly localized in the SAN center, and other surrounding cells such as contractile cardiomyocytes, adipocytes, fibroblasts, and immune cells. This cell heterogeneity imparts complexity to overall SAN structure and function, and the coordination among functions of these cells is important to SAN health. Although a recent study using single nucleus RNA-seq (GSE130710) has identified different types of cell clusters within SAN, the distinct functions of pacemaker cells and other cell types within the SAN tissue have not been delineated.

Added value of this study

We discovered that: (1) the main characteristics of cells within the sinus node myocyte cluster in GSE130710 featured genes that drive the coupled-clock system of pacemaker cells; (2) the expression of genes that regulate fatty acid and nitrogen metabolism and immune signaling was reduced in the sinus node myocyte cluster; (3) the nuclear immuno-labeling of proliferation markers was rare in sinus node myocyte cluster but abundant in non-myocyte interstitial cells; (4) endocardial, epicardial and epithelial cells, and macrophages expressed proliferation-related genes at a higher level than other cell clusters in GSE130710; (5) cells with higher proliferation signals (5.1) displayed negatively enriched fatty acid and nitrogen metabolism pathways, similar to sinus node myocytes, but (5.2) showed positive enrichment in expression of genes that regulate immune signaling, in contrast to sinus node myocytes.

Implications of all the available evidence

Our study discovered that different cell clusters in the heart pacemaker tissue, the SAN, differed in functional capacity, ranging from excitability, metabolism, proliferation, and immune function. These findings expand the understanding of SAN cell functions, from bulk tissue to single nucleus resolution. This elucidation of specific functions of different cells within the heart's pacemakers is critical to the development of novel therapies to treat pathologies affecting the rate and rhythm of the heartbeat.

## 1 Introduction

The heart is a central player within a hierarchical system of clocks operating within the autonomic neurovisceral axis that creates and synchronizes rhythmic functions ranging from milliseconds to days and beyond [1,2]. The heart is composed of different compartments with specialized functions. The sinoatrial node (SAN) initiates the heartbeat, maintains its normal cardiac rhythm, and harbors many different cell types, including pacemaker cells, devoid of myofilaments [3], mainly localized in the center of SAN, and other surrounding cells expressing robust contractile myofilaments [4,5].

The heart's beating rate and rhythm are regulated by autonomic input to SAN pacemaker cells that modulates functions within a coupled-clock system intrinsic to SAN cells [6]. The SAN pacemaker cell coupled-clock system comprised a “calcium clock,” the sarcoplasmic reticulum (SR), which continuously oscillates Ca^2+^; the Ca^2+^ clock is continuously but variably coupled to a “membrane clock,” an ensemble of surface membrane ion channels. The “biochemical engine” of the coupled-clock system is a constitutively active, Ca^2+^ calmodulin-dependent adenylyl cyclase (AC) that generates cyclic AMP (cAMP), leading to modulation of cAMP-gated ion channels, exchange protein directly activated by cAMP (EPAC) signaling, and protein kinase A (PKA) and Calcium/calmodulin-dependent protein kinase II (CaMKII)-dependent kinase activities, mechanisms that regulate intracellular Ca^2+^ levels, Ca^2+^ dynamics and membrane potential within SAN cells [[6], [7], [8], [9], [10], [11]]. For more details see the reference paper [12].

In addition to pacemaker cells, SAN tissue also contains adipocytes, fibroblasts, and immune cells [4,5]. Moreover, additional cell types/subtypes within the SAN, having unknown functions, remain to be discovered [3]. Signaling between these other cell types and pacemaker cells is important to normal SAN functions. However, the large cell type heterogeneity and details regarding their molecular mechanisms are obstacles in our understanding of the fine details of SAN functions.

Emerging single cell RNA sequencing (RNA-seq) technology is a strong tool to delve into organs and tissues, and it has been applied in many scientific fields such as development, cancer, neuroscience and immune system [13]. This new technology has been applied to the heart with respect to cardiac development and composition, cell interactions, and molecular regulations, but mainly on whole heart, left ventricle or induced cardiomyocytes [14]. Two studies have applied this single cell (RNA-seq) technology to the SAN [15,16]. One of the two studies pooled mouse SAN cells and atrial and ventricular (AV) cardiomyocytes [15], and thus SAN data couldn't be separated from AV data. Another study (GSE130710) only included C57 mouse SAN and classified cell types by quantifying mRNA patterns in single nuclei [16]. Although 12 types of cells within SAN tissue were annotated in the second study, it is not clear to what degree the functions of those cell types can be defined by those single nucleus RNA-seq data, because additional functional analyses were lacking.

Here we utilized the public dataset of that study (GSE130710) [16] to perform AUCell analysis to define subgroups of cells, to perform differentially expressed gene (DEG) analysis to identify biomarker genes, and to perform unbiased functional enrichments of Gene Ontology (GO) terms and Kyoto Encyclopedia of Genes and Genomes (KEGG) pathways to deduce cellular and molecular functions. The biomarkers indicating sinus node cells were identified in DEG analysis and their functions were deduced in GO and KEGG enrichments. The AUCell analysis identified two additional groups of cells. One group of cells highly expressing the list of genes collected from bulk cells studies were defined as SAN characteristic cells. There was a large overlap with regard to two respects (1) nuclei per se and (2) their functions, between those sinus node myocytes and these SAN characteristic cells, proving the consistency and complementarity between single nucleus RNA-seq and bulk cell characteristics. This also supported the feasibility of the single nucleus datasets and our analysis methods. The AUCell analysis also identified another group of cells containing two categories of cell types that showed relatively strong proliferation signals: (1) endocardial, epicardial, and epithelial cells, and (2) macrophages. In summary, by utilizing a list of SAN characteristic genes generated in bulk cell studies, we defined a group of SAN characteristic cells in a single nucleus RNA-seq dataset. We identified the specific function of proliferation in addition to SAN pacemaker cell function that has never been shown in mouse SAN.

## 2 Data retrieval

Sinoatrial node single nucleus RNA-seq count matrix and meta data were retrieved from the public database, GEO, with ID (GSE130710). Sinus nodes were isolated from 16 male C57BL/6J mice, 12–14-weeks-old and 25–35 g body weight. Single nuclei from the sinus nodes of every 8 mice were pooled and labeled as H4_S1 and H5_S2. Then single-nucleus libraries were prepared in the Chromium system (10x genomics) using Chromium Single-Cell 3' Reagent Kits (v3 Chemistry). The RNA-sequencing was conducted on a NovaSeq™ 6000 sequencing system (Illumina) using a S1 Reagent Kit (100 cycles). Then the Cell Ranger 3.0 pipeline (10x Genomics, USA) was used to align reads, quantify unique molecular identifiers (UMI) and generate feature-barcode expression matrices. After filtration, the data was log normalized with the LogNormalize () command in Seurat v.3.0.0.9000 R package.

## 3 Methods

### 3.1 Data preprocess

This work utilized the computational resources of the NIH HPC Biowulf cluster (http://hpc.nih.gov). The RNA-seq count matrix and meta data were imported into Partek® Flow® platform (version: 10.0.21.0602). First, we annotated the cells using the cluster annotation information from the meta data. Then we excluded features where value ≤ 0 in at least 99.9% of the nuclei. We set the threshold by learning from the reference paper [17] and tutorial (https://documentation.partek.com/display/FLOWDOC/Analyzing+Single+Cell+RNA-Seq+Data). Of the 27,998 genes, 62.64% passed the filter, so 17,539 genes remained. Excluded features were in Supplemental Table 1. The quality control was visualized via plotting gene counts, expressed features, percentage of mitochondrial counts, and percentage of ribosomal counts in scatter and violin plots. After checking the proportion of different types of cells in the two samples in the dataset, we used the data of the 5357 SAN nuclei in the subsequent analysis. The proportion of one cell type is the percentage of the number of that type of cells in all cells. Proportion (Cell type A) = Absolute number (Cell type A)/Absolute number (All cells) X 100 (%).

### 3.2 Dimension reduction and clustering

Principal component analysis (PCA) was performed using the parameter (feature contribute: by variance). The scree plot showed that the top 20 principal components were sufficient to represent all the features, so the top 20 principal components were used in most downstream analyses to balance the effect and time and resource consumption. We adopted two methods in clustering. The first method, graph-based clustering, produced 13 clusters with the top 20 principal components from PCA. In the second method, k-means clustering with the top 20 principal components, we trained a range of k values (from 3 to 20) and finally selected k = 5 as the best parameter via Compare Clusters function embedded in Partek Flow (version: version: 10.0.21.0602) [18] (Supplementary Figure 4). In either t-distributed stochastic neighbor embedding (t-SNE) or uniform manifold approximation and projection (UMAP), nuclei were colored in three ways: (1) cluster annotation in the reference paper, (2) graph-based cluster in this study, and (3) K-means cluster (k = 5) in this study. To visualize the nuclei after dimension reduction and clustering in two-dimensional plot, we performed t-SNE and UMAP analyses with the top 20 principal components from PCA. We trained two different feature contribution parameters, equally and by variance, in t-SNE analysis and selected equally as the best parameter. We also trained two different output value initialization parameters, random and spectral, in UMAP analysis. Because the clusters were divided more completely and more consistent with those in the reference paper [16] in UMAP within the parameter, random, we selected “random” as the best parameter.

### 3.3 Trajectory analysis

The scaled gene expression values were used in trajectory analysis using Monocle 2. Three branches and the pseudotime were inferred. In the pseudo trajectory, nuclei were colored in four ways: (1) three states in three branches, (2) cluster annotation in the reference paper, (3) graph-based cluster in this study, and (4) K-means cluster (k = 5) in this study.

### 3.4 Gene expression visualization

The expressions of representative genes were visualized in feature plots. Color represents the gene expression level. The brighter the color was, the high level the gene expression was. In addition, AUCell analysis was also used to visualize the gene set expression. The AUCell method uses the "Area Under the Curve" (AUC) to calculate whether a list of genes is enriched within the expressed genes for each nucleus. The distribution of AUC scores across all the nuclei allowed us to infer the similarity of nuclei to the features of the gene lists, including cardiac rhythm and contraction in Ed list and cellular proliferation in proliferation list (Supplementary Tables 2 and 3). The average expression of individual gene in those lists was also shown across each cell type in heatmap, where cell types and genes were clustered in hierarchy.

### 3.5 Differentially expressed gene calculation

Because of the characteristics of single cell or single nucleus RNA-seq data, we selected two statistical methods, ANOVA and Hurdle, to calculate the ratio and FDR of gene expression in one certain group of cells vs. other cells. For example, when we study sinus node myocytes, the comparison was between cells in the cluster of sinus node myocytes and all other cells. Specific comparisons were defined in legend corresponding to each figure. FDR represents false discover rate, the ratio of the number of false positive results to the number of total positive test results. The results were shown in volcano plots. Differentially expressed genes from the two methods were compared in venn diagram and the genes in the intersection were summarized in pie plots. Details were in the first three sheets of Supplementary Table 4-6.

### 3.6 Gene Ontology (GO) terms and Kyoto Encyclopedia of Genes and Genomes (KEGG) pathways enrichment

Partek Flow enabled us to enrich the GO terms and KEGG pathways from a certain group of cells vs. other cells. The default statistical method in Partek Flow, pathway ANOVA (similar to Gene Set Enrichment Analysis (GSEA)), was used to calculate the ratio and FDR between gene expression in one group of cells vs. other cells. The results were shown in volcano plots. From GO term and KEGG pathway lists, the upregulated part and downregulated part were further studied separately. Then GO terms and KEGG pathways are ranked by absolute (log2(Ratio)), from largest to smallest. The top upregulated and downregulated parts were listed separately for GO terms and KEGG pathways. Details were in the last two sheets of Supplementary Table 4-6. Enrichments from sinus nodes cells, cells highly expressing genes in Ed list, and cells highly expressing genes in proliferation list were summarized and compared in Supplementary Table 7.

### 3.7 KEGG pathway visualization

Representative KEGG pathways were selected from the top upregulated and downregulated parts and visualized in Partek Flow. The expressions of genes in those pathways were displayed in different colors. Red and green indicated genes are expressed higher and lower in a certain group of cells vs. other cells respectively. The brighter the color, the larger degree of the ratio of gene expression between a group of cells vs. others.

### 3.8 Immune staining with EdU loading

Male C57BL/6J mice (three months old, 25–28 g, and supplied by Jackson Laboratory) were administered 0.35 mg/L 5-ethynyl-2'-deoxyuridine (EdU) (Santa Cruz, sc284628) for 28 days via drinking water, changed every third day. Mice were administered 60 mg/kg pentobarbital (Sigma P3761) via intraperitoneal injections (IP) Heart was removed and placed in Phosphate-Buffered Saline (PBS) (1X PBS 46-013-CM - Corning). The aorta was cannulated, and the heart was perfused with PBS for 5 min followed by perfusion at ∼100 mmHg with paraformaldehyde (Fisher, 50-980-492) diluted to 4% in PBS, for approximately 10 min or until flow rate was greatly reduced. Hearts were stored in fresh 4% paraformaldehyde for 24 h at 4°. Hearts were washed with PBS and imbedded in 4% low melting point agarose. Sequential transverse sections from the heart of C57BL/6J mice were sectioned on the Leica Vibratome VT1000s from 200 to 300 μM. Sections were permeabilized with 0.2% Triton (Sigma, X100), 23 g/L glycine (Sigma, G7126), and 2% DMSO (Sigma, D8418) for 3 days. EdU labeling (Click Chemistry Tools cat # 1350) was performed followed by staining with 4′,6-diamidino-2-phenylindole (DAPI) 1:300 (Sigma, D9542) and HCN4 1:500 (Alomone labs, APC-052). Microscopic fields were visualized in cardiomyocytes via fluorescent imaging (Zeiss LSM 980) at 400× magnification. All studies were performed in accordance with the Guide for the Care and Use of Laboratory Animals published by the National Institutes of Health (NIH Publication no. 85-23, revised 1996). The experimental protocols were approved by the Animal Care and Use Committee of the National Institutes of Health (protocol #441-LCS-2016).

## 4 Result

### 4.1 Profiling of gene expression in single nuclei in mouse sinoatrial node

To profile the gene expression in single sinoatrial node (SAN) cells, we customized the analysis of the single nucleus RNA-sequencing (RNA-seq) dataset with ID (GSE130710) [16] within the Partek® Flow® platform as described in Method. Both the counts of each gene (feature) (Supplementary Figure 1a) and detected features (genes) in each nucleus (Supplementary Figure 1b) were concentrated round the median. Because neither mitochondrial counts (Supplementary Figure 1c) nor ribosomal counts (Supplementary Figure 1d) occupied a large proportion of the total counts, indicating the absence of too many dead cells or aggregated cells, the filtered data met the criteria for subsequent analyses. Next, we summarized the cell type proportion in the two pooled SAN samples. There was a large heterogeneity in the SAN samples. Compared with the first sample (H4_S1), more sinus node myocytes were identified in the second sample (H5_S2) (Supplementary Figure 2).

**Figure 1:**
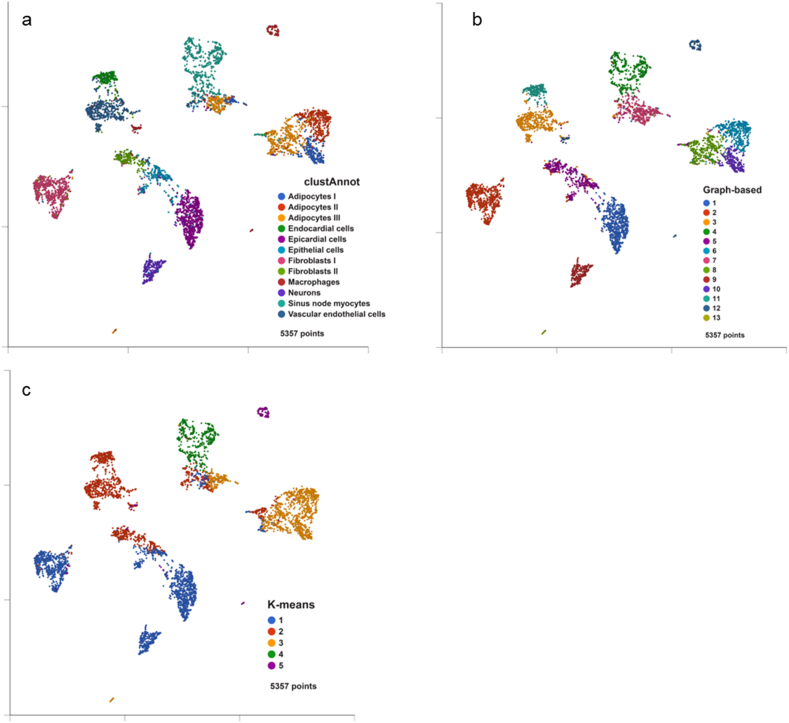
**Uniform manifold approximation and projection (UMAP) analysis.** The single nuclei were displayed in UMAP with the first 20 principal components and colored by cluster annotation in the reference paper (a), graph-based cluster in this study (b), and K-means cluster (k = 5) in this study (c).

Due to the enormous number of nuclei and a great number of features (genes) identified in the dataset, we first performed the principal component analysis (PCA) to reduce the dimension of features (Supplementary Figure 3), and then clustered the sample nuclei using two methods (graph-based and K-means clustering (k = 5, Supplementary Figure 4)). The nuclei were clustered into 13 and five clusters by the two methods, respectively. To better visualize the nuclei in a 2-D space, we conducted the T-distributed stochastic neighbor embedding (t-SNE) analysis with the top 20 principal components and colored the nuclei using three methods: cluster annotation [16] (Supplementary Figure 5a), graph-based cluster (Supplementary Figure 5b), and K-means cluster (k = 5) (Supplementary Figure 5c). In the first method (Supplementary Figure 5a), nuclei were clustered and located in the t-SNE plot consistent with the result in the reference paper [16]. Because nuclei could not be separated from each other very clearly in t-SNE, we employed an additional method, uniform manifold approximation and projection (UMAP) (Figure 1), to visualize the clustering of nuclei, colored in the same fashion as those in supplementary figure 5. The graph-based cluster method (Figure 1b and Supplementary Figure 5b) had the similar effect to the cluster annotation method used in the reference paper [16] (Figure 1a and Supplementary Figure 5a), while the K-means cluster method (Figure 1c) combined some cell types sharing the similar gene expression into the same cluster, e.g., most Fibroblasts I, Epithelial cells, and neurons into cluster 1, and most Adipocytes I, II, and III into cluster 3.

**Figure 2:**
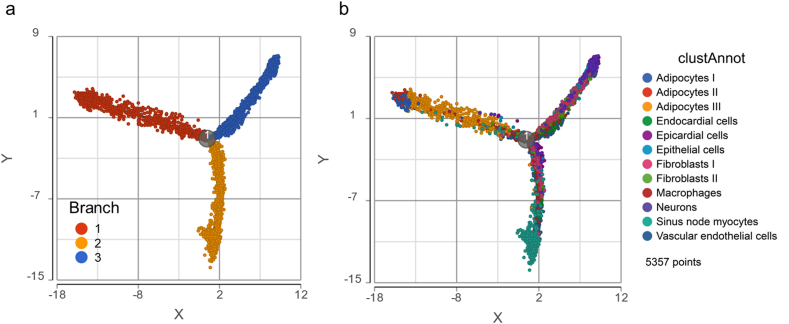
**Trajectory analysis in Monocle 2.** (a) Three branches in the trajectory were inferred in Monocle 2. (b) The single nuclei were also colored by cluster annotation in the reference paper.

We utilized Monocle 2 to deduce the pseudotime trajectory [19] of the SAN cells containing the nuclei used in this dataset. Of note, three branches representing three cell states were generated (Figure 2a). Shown with cluster annotations, the state 1 branch mainly contained adipocytes I, II, and III; the state 2 contained macrophages, neurons, vascular endothelial cells, and sinus node myocytes; the state 3 contained endocardial cells, fibroblasts I and II, epithelial cells, and epicardial cells (Figure 2b). The distribution of adipocytes, epicardial cells, and sinus node myocytes in the termini of the three branches suggested that they are three completely different cell types (Figure 2b). The pseudotime trajectories colored by clusters from graph-based (Supplementary Figure 6a) and k-means (Supplementary Figure 6b) clustering methods also supported this suggestion.

### 4.2 Characterization of nuclei of sinus node myocytes vs. other cells within the SAN

**Figure 3:**
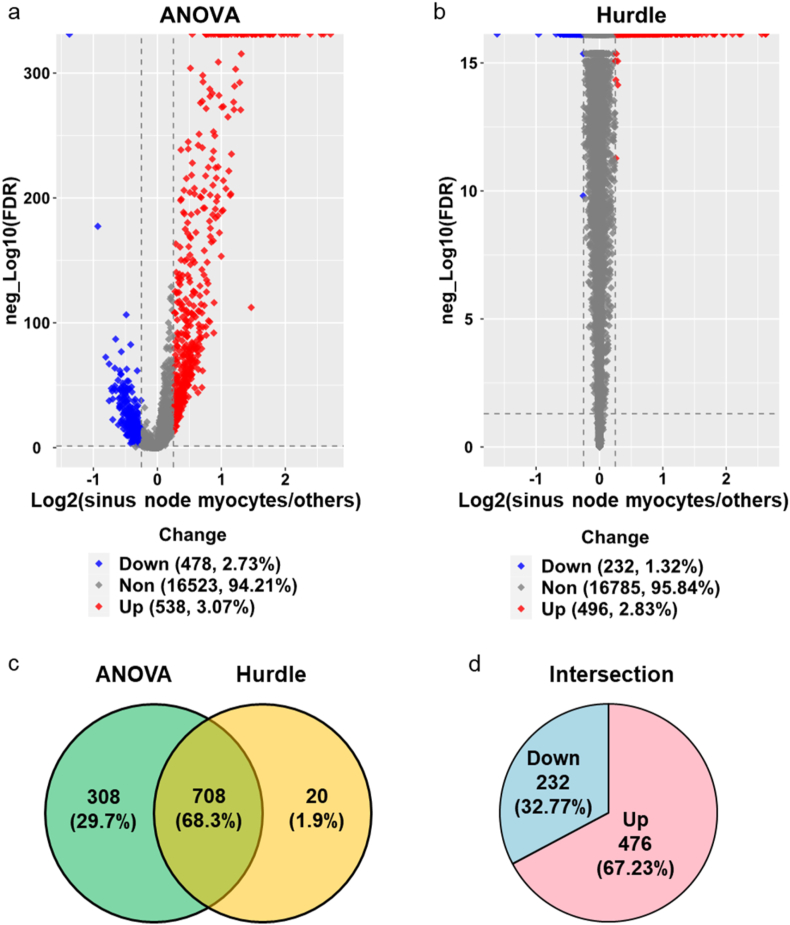
**Differentially expressed genes in nuclei of sinus node myocytes vs. other cells.** Volcano plots of the differentially expressed genes obtained using two statistical methods, ANOVA (a) and Hurdle (b), and their venn diagram (c). The regulation directions of genes in the intersection of (c) are shown in pie plot (d). The cutoff is: -log10(FDR) > 1.3, and absolute (log2(Ratio)) ≥ 0.25. The ratio is between sinus node myocytes and other cells. FDR represents false discover rate.

To explore the distinct functions of sinus node myocytes from other cell types, we compared the gene expression and gene set enrichments within this cluster of sinus node myocytes to those within other cell clusters identified in SAN. We adopted two appropriate statistical methods, ANOVA and Hurdle models, in differentially expressed gene (DEG) analysis to assess the gene expression differences between nuclei of sinus node myocyte cluster and those within other SAN clusters (Figure 3a and b). The results obtained from the two methods indicated that 708 genes significantly differed in the same direction between sinus node myocyte cluster and other SAN cell clusters (Figure 3c), and of these 708 genes, the number of upregulated genes was twice that of downregulated genes (Figure 3d). The complete list of genes was in the supplementary Table 4, and those DEGs could be used as biomarkers between these two types of cells in future studies.

**Figure 4:**
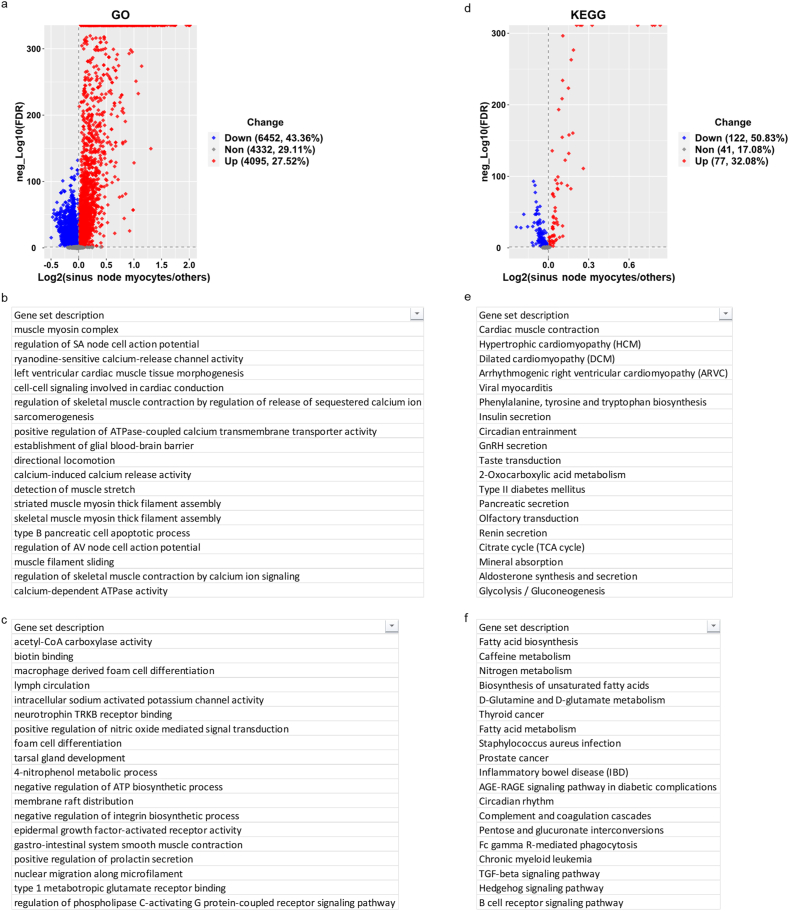
**Differentially enriched GO terms and KEGG pathways in nuclei of sinus node myocytes vs. other cells.** Volcano plots of the differentially enriched GO terms (a) and KEGG pathways (d). The cutoff is: -log10(FDR) > 1.3, and absolute (log2(Ratio)) > 0. Then GO terms and KEGG pathways are ranked by absolute (log2(Ratio)), form largest to smallest. The top GO terms enriched from upregulated and downregulated genes in sinus node myocytes vs. other cells are listed in (b) and (c) respectively. The top KEGG pathways enriched from upregulated and downregulated genes in nuclei of sinus node myocytes vs. other cells are listed in (e) and (f) respectively. The ratio is between sinus node myocytes and other cells. FDR represents false discover rate.

**Figure 5:**
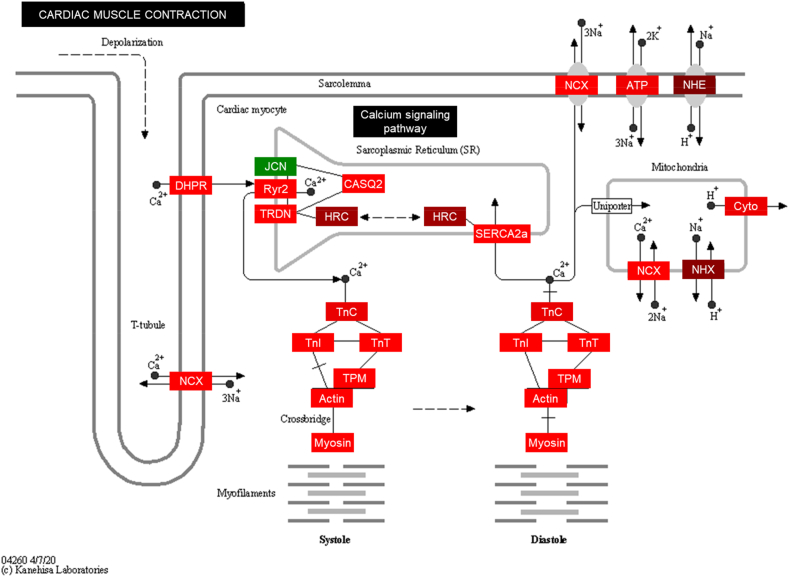
**Visualization of the KEGG pathway, cardiac muscle contraction, with differentially expressed genes in nuclei of sinus node myocytes vs. other cells.** One of the representative KEGG pathways, cardiac muscle contraction, is visualized. Color shows cell typic difference in gene expression between sinus node myocytes and other cells. Red and green indicate genes expressed higher and lower in nuclei of sinus node myocytes compared to other cells respectively.

In addition to the DEG analysis, we adopted the pathway ANOVA, which is the principle behind Pathway in Partek® Flow®, to reveal pathways in which several genes may only be slightly upregulated but have profound biological consequences. In brief, expression values of all the genes within a gene set (e.g., a Gene Ontology (GO) term or Kyoto Encyclopedia of Genes and Genomes (KEGG) pathway gene set) were added up and then those sums were compared between the samples/groups (Figure 4a and d). Then the enriched GO terms and KEGG pathways were separated into two parts, significantly positively enriched and negatively enriched gene sets. We listed the top gene sets ranked by absolute values of log2FoldChange of all gene sets whose FDR values were less than 0.05, i.e., -log10(FDR) > 1.3 (Figure 4b, c and e, f). The results from GO and KEGG enrichments were highly consistent. Cardiac contraction related functions were deduced from the GO and KEGG enrichments (Figure 4b and e), as would have been expected in a cluster of cells labeled as sinus node myocytes in the GEO database [16]. We next compared the deduction of Figure 5 to the functions of SAN. Figure 5 displayed the cardiac muscle contraction KEGG pathway. As stated in the KEGG website, cardiac muscle contraction is a complex process, initiated by membrane depolarization, transduced by Ca^2+^ signaling pathway in sarcoplasmic reticulum and ion exchanges on cellular and mitochondrial membrane, activated by Ca^2+^ binding to troponin in myofilaments. Almost all components in this process/pathway were expressed higher in sinus node myocytes than other cells (Figure 5). The enrichments depicted, in fact, pointed to genes that had already been verified as being crucial to excitation-contraction-relaxation-coupling in cardiac sinus node pacemaker cells [12,16,20]. Therefore, the bioinformatic analysis deduction from information generated within the single nucleus RNA-seq data of SAN tissue are correct, and thus validate the method of deriving cell functions from single nucleus RNA-seq data, e.g., genes within the KEGG pathway diagram (red and green indicate upregulation or downregulation respectively) in nuclei of sinus node myocytes (Figure 5). Clearly, numerous molecules involved in excitation, contraction, relaxation, coupling in cardiac myocytes, except for JCN (junctin, aspartate beta-hydroxylase) were upregulated in nuclei of sinus node cells compared with other cells.

In addition to those functions depicted in Figure 5, GO and KEGG analyses of the sinus node myocyte cluster with SAN tissue pointed to negative regulation of numerous functions. For example, in GO analysis, fatty acid metabolism and nitrogen metabolism were negatively enriched in sinus node myocytes, supported by the negative enrichments of GO molecular function term, such as acetyl-CoA carboxylase activity, GO biological process term, such as positively regulation of nitric oxide mediated signal transduction (Figure 4c). Similarly, numerous pathways were negatively enriched in KEGG analysis, such as fatty acid biosynthesis, biosynthesis of unsaturated fatty acids, fatty acid metabolism, and nitrogen metabolism (Figure 4f). In addition, some immune signaling KEGG pathways were also negatively enriched, e.g., AGE-RAGE signaling pathway, TGF-beta signaling pathway, hedgehog signaling pathway, B cell receptor signaling pathway, etc. (Figure 4f), pointing to the idea that the immune functions are suppressed within and among cells comprising the cell cluster designated as sinus node myocytes within SAN tissue [16].

**Figure 6:**
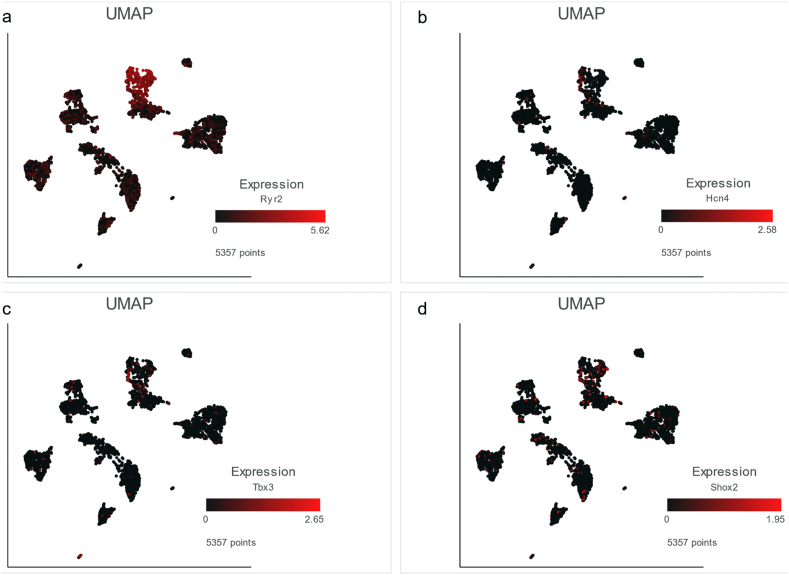
**Expression of representative genes in single nucleus.** Feature plots show the expression of Ryr2 (a), Hcn4 (b), Tbx3 (c) and Shox2 (d) in the projection of UMAP. The redder the color is, the higher the gene expression is in the single nucleus.

Now that we had the single nucleus data and annotated cell types, in order to reduce the bias of cell type annotation in the reference paper [16], we sought to compare the results from single nucleus and our low-throughput experiments to ascertain the major cell types responsible for SAN functions by analyzing a list of genes collected by our lab (Ed list in Supplementary Table 2). Our lab has been studying excitation, Ca^2+^ cycling and contraction for several decades. Our studies focus on both biochemical and biophysical mechanisms. Ed list is an overview of many of the molecules that are known to be important in excitation, Ca^2+^ cycling and contraction in cardiac cells [[20], [21], [22], [23], [24]]. The results confirmed the consistent characteristics of sinus node myocytes (Supplementary Figure 7-9). Previously, four genes, Ryr2 (ryanodine receptor 2, cardiac), Hcn4 (hyperpolarization-activated, cyclic nucleotide-gated K^+^ 4), Tbx3 (T-box 3), and Shox2 (short stature homeobox 2), were regarded as SAN marker genes [[25], [26], [27], [28]], but only Ryr2 was in the intersection and significantly upregulated in the nuclei of SAN characteristic cells (Supplemental Table 4). To verify this result, we generated feature plots and observed the dispersion of the four genes in SAN, but only the expression of Ryr2 was particularly high in the group of SAN characteristic cells or sinus node myocytes (Figure 6a–d). Based on our analyses, Ryr2 was expressed markedly higher in sinus node myocytes than in other SAN cell types. By combination of Ryr2's important roles in SAN [28], we proposed Ryr2 as a candidate marker to study myocytes inside SAN when comparing with other cell types inside SAN. We admitted the limitation in this study that we only had the nucleus RNA data which may miss much information in the cytoplasm, so we set a constraint in studying nucleus. Therefore, it is Ryr2, rather than Hcn4, Tbx3 or Shox2, that can be used as the candidate SAN marker gene inside nucleus, especially when studying the SAN characteristic cells or sinus node myocytes among heterogeneous SAN cells.

### 4.3 Characterization of nuclei of proliferative cells

**Figure 7:**
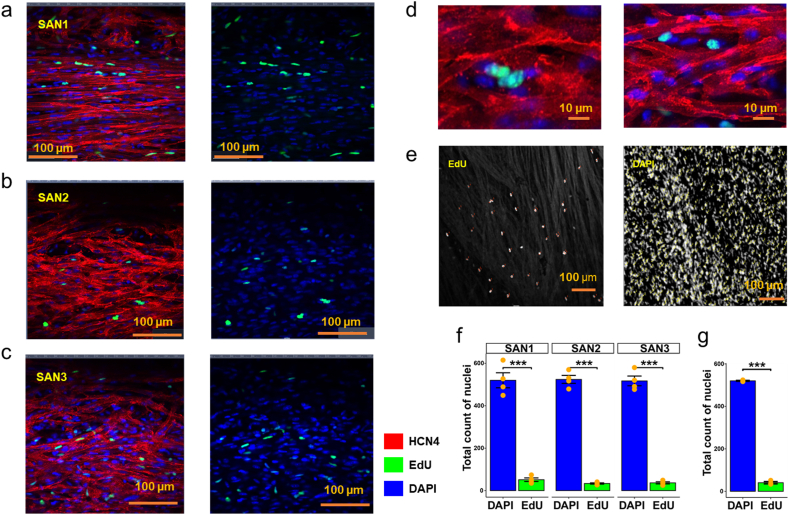
**Immune staining with EdU loading.** Evidence of proliferating cells in SAN region in C57BL/6J mice. (a,b,c) Representative examples from three individual mice of HCN4 positive regions with EdU and DAPI stainings (20x). Overlapping of green EdU nuclei with blue DAPI nuclei are visible as blue green. (d) Examples of EdU positive regions in higher magnification illustrate overlapping of EdU (green) and DAPI (blue) signals, detected only within interstitial space between HCN4 positive (red) pacemaker cells. (e) Images illustrate counting of EdU+ (left) DAPI + (right) nuclei within SAN preparations (20x). (f, g) Bar plots with dots and error bars (SEM) of total count of nuclei. (f) Each dot represents an area randomly selected within each SAN preparation. Y-axis denotes count of EdU+/DAPI + nuclei (green) and EdU-/DAPI + nuclei (blue). (g) Each dot represents each SAN (SAN1, SAN2 and SAN3). Y-axis denotes average count in four areas of each SAN. Paired sample *t*-test was used. *** indicates p-value <0.001.

**Figure 8:**
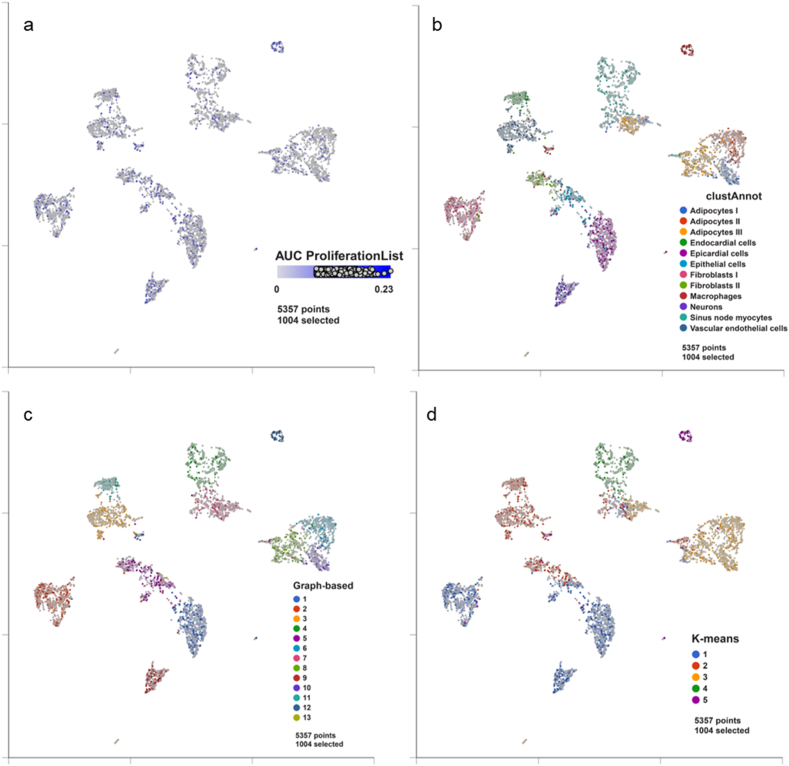
**Expression of proliferation list genes in nuclei of all types of SAN cells.** Top nuclei expressing gene in the proliferation list are displayed in feature plots in the projection of UMAP and colored by AUCell score (a), cluster annotation in the reference paper (b), graph-based cluster in this study (c), and K-means cluster (k = 5) in this study (d).

Although cell proliferative and regenerative characteristics of cells that reside in the heart's SAN are crucial to heart health [16], most studies have examined cell proliferative capacity in ventricular and atrial cells [29–40], and little is known about cell proliferation within the SAN. Our approach was to directly visualize cellular DNA synthesis in S phase of cells in the SAN utilizing EdU [41]. To this end, we loaded EdU into three-month old C57BL/6J mice to identify nuclear labeling of EdU within cells in intact SAN tissue. Confocal images indicated that nuclear uptake of these proliferation markers occurred in interstitial cells but not in SAN pacemaker cells (Figure 7a–g). We next investigated the cell proliferation characteristics within the single nucleus dataset (GSE130710) [16]. There was not a very clear aggregation of nuclei displaying high proliferation signals as shown in the AUCell scores in Figure 8a. Because different cell types are assumed to differentially contribute to cardiac regeneration [42], we carefully examined proliferation signals (proliferation AUCell scores) in each cell type, cells with proliferative characteristics were sparsely distributed in the sinus node myocyte cluster but widely distributed among other cell clusters within the single nucleus dataset (GSE130710) [16]. There were two categories of proliferative cell types within the UMAP that showed relatively strong proliferation signals. One category consisted of endocardial, epicardial, and epithelial cells (Figure 8b–d), in line with many previous studies of those cell types in ventricle or atria regeneration [[29], [30], [31], [32], [33],^36^]. Macrophages were the second category of cells within the SAN (Figure 8b–d) showing strong proliferation signals (57.71% of macrophages) (Supplemental Table 3, Sheet 3). The locations of macrophages (near the fork) and sinus node myocytes (in the terminus) in the same branch of the pseudotime trajectory even indicated the potential regeneration of sinus node myocytes from macrophages (Figure 2). Our results and previous discoveries that inflammation and immune cells like macrophages [[34], [35], [36],^40^] were involved in myocytes regeneration in atrial, atrioventricular nodal, and ventricular cells support the idea that constitutive inflammation accompanies cell proliferation within the SAN.

**Figure 9:**
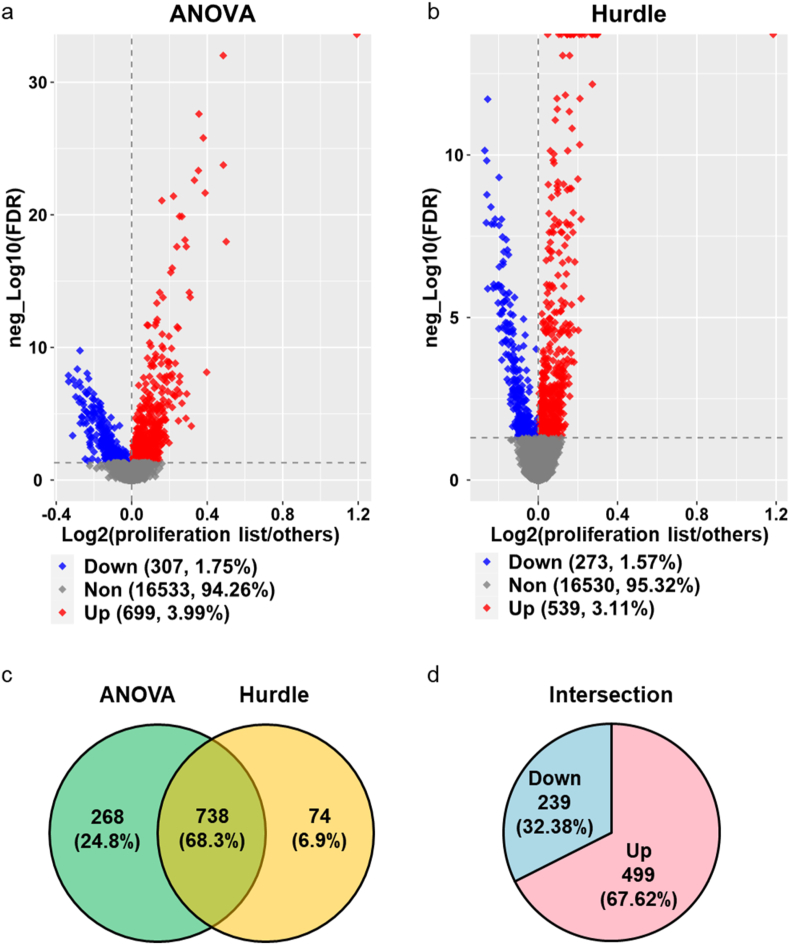
**Differentially expressed genes in nuclei of proliferation list cells vs. other cells.** Volcano plots of the differentially expressed genes obtained using two statistical methods, ANOVA (a) and Hurdle (b), and their venn diagram (c). The regulation directions of genes in the intersection of (c) are shown in pie plot (d). The cutoff is: -log10(FDR) > 1.3, and absolute (log2(Ration)) > 0. The ratio is between proliferation list cells and other cells. FDR represents false discover rate.

To unravel the molecular mechanisms underlying proliferation and regeneration of the SAN cells, we next conducted ANOVA and Hurdle model analyses to discover the DEGs specific to nuclei of cells with high proliferation AUCell scores (proliferation list cells) compared with other SAN cells within the dataset (GSE130710) [16] (Figure 9a and b). We obtained 738 concordantly regulated genes in the ANOVA and Hurdle models (Figure 9c), among which 499 genes were upregulated in proliferative cells dispersed throughout the cell clusters (Figure 9d).

**Figure 10:**
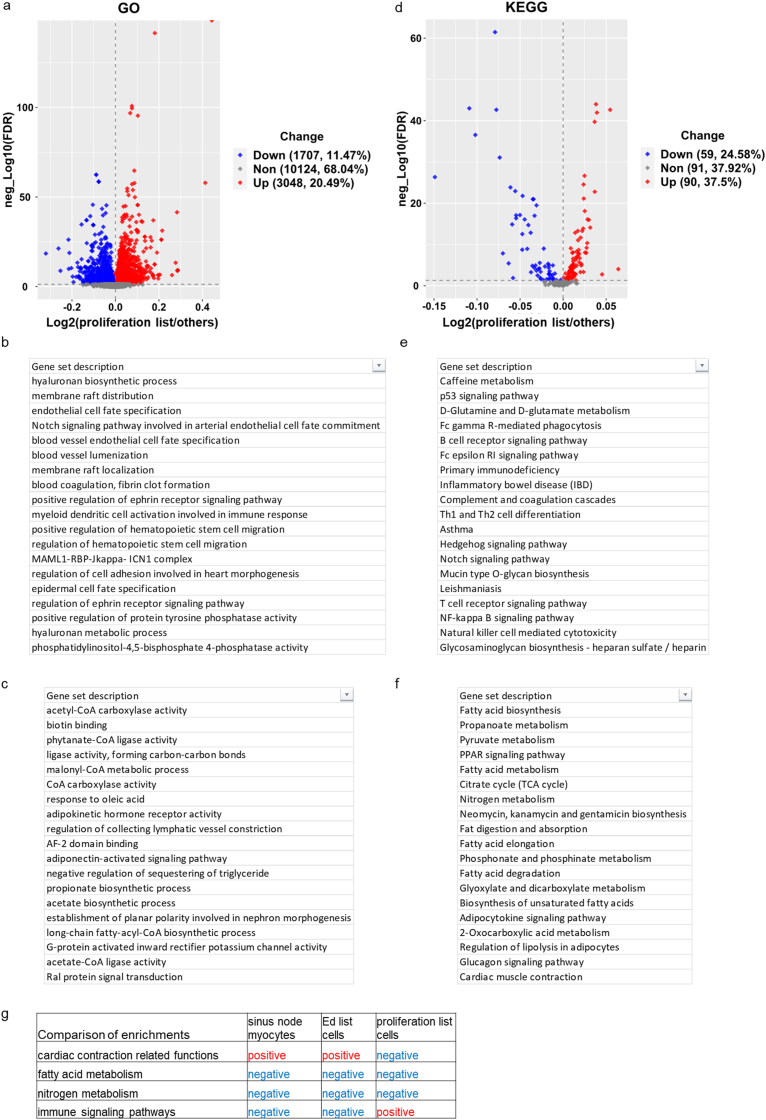
**Differentially enriched GO terms and KEGG pathways in nuclei of proliferation list cells vs. other cells.** Volcano plots of the differentially enriched GO terms (a) and KEGG pathways (d). The cutoff is: -log10(FDR) > 1.3, and absolute (log2(Ratio)) > 0. Then GO terms and KEGG pathways are ranked by absolute (log2(Ratio)), form largest to smallest. The top GO terms enriched from upregulated and downregulated genes in sinus node myocytes vs. other cells are listed in (b) and (c) respectively. The top KEGG pathways enriched from upregulated and downregulated genes in nuclei of proliferation list cells vs. other cells are listed in (e) and (f) respectively. g. Comparison of enrichments from sinus node myocytes, Ed list cells, and proliferation list cells. The ratio is between proliferation list cells and other cells. FDR represents false discover rate.

We continued to study the GO terms (Figure 10a–c) and KEGG pathways (Figure 10d–f) showing the characteristics of these proliferative cells. On the one hand, there was a striking similarity of the characteristics of these proliferative cells and cells within sinus node myocyte cluster. Negative enrichment of fatty acid metabolism and nitrogen metabolism common to both proliferative cells and those within sinus node myocyte cluster included: GO terms (acetyl-CoA carboxylase activity, phytanate-CoA ligase activity, malonyl-CoA metabolic process, CoA carboxylase activity, adipokinetic hormone receptor activity, adiponectin-activated signaling pathway, negatively regulation of sequestering of triglyceride, propionate biosynthetic process, long-chain fatty-acyl-CoA biosynthetic process, etc.) (Figure 10c); and KEGG pathways (fatty acid metabolism (Supplementary Figure 10), fatty acid biosynthesis, propanoate metabolism (Supplementary Figure 11), PPAR signaling pathway (Supplementary Figure 12), fat digestion and absorption, fatty acid elongation, fatty acid degradation, biosynthesis of unsaturated fatty acids, adipocytokine signaling pathway, 2-Oxocarboxylic acid metabolism, regulation of lipolysis, nitrogen metabolism, etc.) (Figure 10f).

On the other hand, proliferative cells manifested some characteristics that differed from those within sinus node myocyte cluster, including: immune signaling pathways were positively enriched, e.g., KEGG pathways (p53 signaling pathway (Supplementary Figure 13), Fc gamma R-mediated phagocytosis (Supplementary Figure 14), B cell receptor signaling pathway (Supplementary Figure 15), Th1 and Th2 cell differentiation, Hedgehog signaling pathway, Notch signaling pathway, T cell receptor signaling pathway, natural killer cell mediated cytotoxicity, etc.) (Figure 10e). In contrast, cardiac muscle contraction processes that were markedly positively enriched in the sinus node myocyte cluster were negatively enriched in proliferative cells (Figure 10f).

Taken together, we identified two categories of proliferative cell types within single nucleus RNA-seq dataset (GSE130710) [16]: endocardial, epicardial, and epithelial cells; and macrophages, suggesting that chronic proliferation of these cell types occurs within SAN tissue.

## 5 Discussion

It is acknowledged that heterogeneous cells constitute SAN [4,5]. We used the Partek® Flow® and R language to perform in-depth functional analysis of cells that had been identified in the single nucleus dataset (GSE130710) [16]. Following dimension reduction, we clustered the nuclei in distinct cell types using t-SNE or UMAP methods (Figure 1 and Supplementary Figure 5). More interestingly, the nuclei were inferred to follow a pseudotime trajectory, in which three categories of cells in the termini of three branches were: (1) adipocytes, (2) sinus node myocytes, (3) endocardial, epithelial, and epicardial cells (Figure 2).

We next used GO and KEGG enrichment analyses to characterize different biological features of myocytes (in the yellow branch) and proliferative cells (in the yellow and blue branches), that may point to their functions within the intact SAN tissue.

### 5.1 SAN myocyte functions

Cells in the second terminal category (sinus node myocyte cluster) exhibited the major characteristics of SAN pacemaker cells (Supplementary Figure 7-9), demonstrated previously in single cell functional analysis and cardiac contraction related functions [6,43]. Interestingly, the overall expression of Hcn4 within the sinus node myocyte cluster was not as strong as expected (Figure 6), given that Hcn4 channels are crucial players in the coupled clock system that drives SAN pacemaker cell function. Although the weak signal of Hcn4 could be due to a limitation of isolation of single nuclei instead of intact cells or the method of deriving the single nucleus clusters [16], it may indicate that only a minority of Hcn4 expressing pacemaker cells are required to drive SAN pacemaker function [44].

Unexpectedly, fatty acid metabolism, nitrogen metabolism, and immune responses were *negatively* enriched in sinus node myocytes cluster, suggesting that fatty acid and nitrogen metabolism, and cytokine/chemokine production might differ in SAN pacemaker cells compared to other cell types within the SAN. Indeed, it has been shown that Aldoc (aldolase c) and aerobic glycolysis, rather than tricarboxylic acid cycle, pyruvate oxidation, and fatty acid catabolism, promoted beating rates in SANs [45].

### 5.2 Proliferative cell functions

Although contractile atrial and ventricular cardiac myocytes are thought to have limited regeneration potential, there is little information regarding regeneration potential of SAN cell components [37–39]. AUCell analysis discovered two categories of cells expressing proliferation-related genes at higher level than in other cells: endocardial, epicardial, and epithelial cells, and macrophage. Both of the first category (endocardial, epicardial, and epithelial cells) [29–33] and the second category (macrophage) [[34], [35], [36],^40^], have been reported to contribute to myocytes regeneration in atrial, atrioventricular (AV) nodal, and ventricular cells, but to our knowledge, there is no previous report for SAN tissue. We further characterized these SAN tissue cells by DEG, GO terms and KEGG pathways. Consistent with nuclei within the sinus node myocyte cluster, in which cardiac muscle contraction was enriched, fatty acid and nitrogen metabolism were *negatively* enriched in proliferative cells, whereas immune signaling pathways were *positively* enriched in proliferative cells (Figure 10g). Therefore, the negatively regulated fatty acid metabolism and positively regulated immunity system in proliferative cells may be crucial for proliferation of these cells at the cost of losing their contraction capability. The proliferative capacity of the immune cells may create an environment required for non-committed cells to regenerate prior to their differentiation into other cell types [46]. In this regard, macrophages, which play an important role in inflammation, are involved in regeneration of ventricular or AV myocytes [38,39].

### 5.3 Limitations

Only two reports have been published on mouse SAN. One paper pooled atrial and ventricular myocytes and SAN cells [15], so that data specific to SAN can not be identified. Another paper applied the single cell nucleus RNA-seq technique rather than single intact cell RNA-seq technique to study all cell types in SAN [16]. We made use of the single nucleus RNA-seq dataset (GSE130716) [16] and applied bioinformatic analysis to analyze all cell nuclei in SAN. Similarly with results in Ref. [15], that cluster 1 represented pacemaker cells highly expressing the marker gene, Ryr3, our study identified Ryr2 as the marker gene in the pacemaker cell cluster, i.e., sinus node myocyte cluster. However, the single nucleus RNA-seq technique fails to identify many cytoplasmic RNAs. In addition, there were only two pools of samples in that study [16] which produced a small number of cells. Further, the analysis was derived from SAN tissue biopsies, which may have been contaminated by the inclusion of atrial tissue, possibly introducing a bias in favor of genes highly expressed in atria.

Previously, bulk RNA-seq and proteomics were used to study SAN characters [16,47]. Both of the two studies compared mouse SAN with right atria (RA). The bulk RNA-seq study identified a new SAN pacemaker cell maker, Isl1 (Islet-1), in addition to other well-known SAN markers, such as Hcn4, Tbx3, and Shox2 [47]. Differently, we compared among the heterogenous cell types inside SAN. We didn't observe strong signals of Isl1, Hcn4, Tbx3, or Shox2 in pacemaker cells’ nuclei inside SAN. The proteomic study observed that fatty acid metabolism was enriched more robustly in SAN than in RA via over-representative analysis (ORA) with differentially expressed proteins between SAN and RA [16], whereas we observed that fatty acid metabolism was negatively enriched in pacemaker cells compared with other cell types inside SAN via gene set enrichment analysis (GSEA) with all genes ranked by ratio of expression in pacemaker cells vs. others. In addition to the differences in experiment designs (comparison of heterogenous cell types inside SAN vs. comparison between SAN and RA), sampling ways (nuclei vs. intact cells), and analysis methods (GSEA vs. ORA) between our study and previous studies, the discrepancies between our results and those in previous studies can be also derived from the above-mentioned limitations, i.e., weak signals of gene transcripts localized inside nuclei and the possible contamination in the original dataset.

To overcome the limitations, increase the recovery rate of cells in SAN and identify a large number of SAN targets, we plan to isolate SAN cells in the future to conduct single cell RNA-seq in the hope of performing a more comprehensive evaluation of SAN cell types. A study of this sort will not only verify the results and conclusions of the present study but will also reveal more information linked to specific cellular mechanisms. To address another limitation of the present study that the only validation of bioinformatic results was immune labeling in SAN, more in-depth validation will be required in future studies.

In summary, both clustering and pseudotime trajectory analyses strongly suggested that cells within the sinus node myocytes cluster have functions that differed from other clusters. As expected, cardiac contraction related functions were enriched in the sinus node myocyte cluster. Specifically, the enriched molecules pointed to genes that had already been verified as being crucial to excitation-contraction-relaxation-coupling in cardiac sinus node pacemaker cells [12,16,20]. Our analysis also pointed to negative regulation of numerous functions within the sinus node myocyte cluster. For example, fatty acid metabolism and nitrogen metabolism, and immune signaling pathways (AGE-RAGE signaling pathway, TGF-beta signaling pathway, hedgehog signaling pathway, and B cell receptor signaling pathway) were negatively enriched in sinus node myocytes [16]. Finally, we also identified two categories of proliferative cell types [16], (1) endocardial, epicardial, and epithelial cells, and (2) macrophages, consistent with chronic proliferation of non-cardiac myocytes [24]. Thus, our results expand the understanding of mouse SAN at single nucleus resolution and provides a segue for more in-depth future mechanistic studies.

## Data sharing statement

RNA-seq data are available indefinitely at NCBI GEO: GSE130710 (https://www.ncbi.nlm.nih.gov/geo/query/acc.cgi?acc=GSE130710).

## Declarations

### Author contributions statement

Jia-Hua Qu: Conceived and designed the experiments; Analyzed and interpreted the data; Wrote the paper.

Richard Telljohann, Rostislav Byshkov: Performed the experiments.

Edward G. Lakatta: Conceived and designed the experiments; Wrote the paper.

### Funding statement

Edward G. Lakatta was supported by the Intramural Research Program of National Institute on Aging, National Institutes of Health, United States.

### Data availability statement

Data associated with this study has been deposited at GEO database under the accession number GSE130710.

### Declaration of interest's statement

The authors declare no competing interests.

### Additional information

No additional information is available for this paper.

## Appendix A Supplementary data

Supplementary_Figures **Supplementary Figure 1. Quality control.** Gene counts (a), expressed features (b), percentage of mitochondrial counts (c), and percentage of ribosomal counts (d) were displayed in scatter and violin plots. **Supplementary Figure 2. Cell type proportion.** Proportion of different types of cells that were annotated in the reference paper were displayed in stack bar plot (a) and pie plot (b). **Supplementary Figure 3. Dimension reduction in PCA.** Three-dimensional PCA plot of single nuclei in the two samples were displayed in (a), and their eigen values were shown in scree plot (b). x-axis represents the first principal component, y-axis represents the second principal component, and z-axis represents the third principal component. Nuclei from two samples were in two different colors, blue or red. **Supplementary Figure 4. Selection of best K parameter for K-means clustering.** The value, k=5, corresponding to the smallest Davies-Bouldin index is the best k parameter. **Supplementary Figure 5. T-distributed stochastic neighbor embedding (t-SNE) analysis.** The single nuclei were displayed in t-SNE with the first 20 principal components and colored by cluster annotation in the reference paper(a), graph-based cluster in this study (b), and K-means cluster (k = 5) in this study (c). **Supplementary Figure 6. Trajectory colored by clusters produced in graph-based and k-means clustering.** The single nuclei were also colored by graph-based cluster in this study (a) and K-means cluster (k = 5) in this study (b). **Supplementary Figure 7. Expression of Ed list genes in nuclei of all types of SAN cells.** Top nuclei expressing gene in the Ed list are displayed in feature plots in the projection of UMAP and colored by AUCell score (a), cluster annotation in the reference paper (b), graph-based cluster in this study (c), and K-means cluster (k = 5) in this study (d). E. Heatmap of expression of Ed list genes and hierarchy clustering of cell types and genes. **Supplementary Figure 8. Differentially expressed genes in nuclei of Ed list cells vs. other cells.** Volcano plots of the differentially expressed genes obtained using two statistical methods, ANOVA (a) and Hurdle (b), and their venn diagram (c). The regulation directions of genes in the intersection of (c) are shown in pie plot (d). The cutoff is: -log10(FDR) < 1.3, and absolute(log2(Ratio)) ≥ 0.25. The ratio is between Ed list cells and other cells. FDR represents false discover rate. **Supplementary Figure 9. Differentially enriched GO terms and KEGG pathways in nuclei of Ed list cells vs. other cells.** Volcano plots of the differentially enriched GO terms (a) and KEGG pathways (d). The cutoff is: -log10(FDR) < 1.3, and absolute(log2(Ratio)) > 0. Then GO terms and KEGG pathways are ranked by absolute(log2(Ratio)), form largest to smallest. The top GO terms enriched from upregulated and downregulated genes in sinus node myocytes vs. other cells are listed in (b) and (c) respectively. The top KEGG pathways enriched from upregulated and downregulated genes in nuclei of sinus node myocytes vs. other cells are listed in (e) and (f) respectively. The ratio is between Ed list cells and other cells. FDR represents false discover rate.**Supplementary Figure 10. Visualization of the KEGG pathway, fatty acid metabolism, with differentially expressed genes in nuclei of proliferation list cells vs. other cells.** One of the representative KEGG pathways, fatty acid metabolism, is visualized. Color shows cell typic difference in gene expression between sinus node myocytes and other cells. Red and green indicate genes expressed higher and lower in nuclei of proliferation list cells compared to other cells respectively. **Supplementary Figure 11. Visualization of the KEGG pathway, propanoate metabolism, with differentially expressed genes in nuclei of proliferation list cells vs. other cells.** One of the representative KEGG pathways, propanoate metabolism, is visualized. Color shows cell typic difference in gene expression between sinus node myocytes and other cells. Red and green indicate genes expressed higher and lower in nuclei of proliferation list cells compared to other cells respectively. **Supplementary Figure 12. Visualization of the KEGG pathway, PPAR signaling pathway, with differentially expressed genes in nuclei of proliferation list cells vs. other cells.** One of the representative KEGG pathways, PPAR signaling pathway, is visualized. Color shows cell typic difference in gene expression between sinus node myocytes and other cells. Red and green indicate genes expressed higher and lower in nuclei of proliferation list cells compared to other cells respectively. **Supplementary Figure 13. Visualization of the KEGG pathway, p53 signaling pathway, with differentially expressed genes in nuclei of proliferation list cells vs. other cells.** One of the representative KEGG pathways, p53 signaling pathway, is visualized. Color shows cell typic difference in gene expression between sinus node myocytes and other cells. Red and green indicate genes expressed higher and lower in nuclei of proliferation list cells compared to other cells respectively. **Supplementary Figure 14. Visualization of the KEGG pathway, Fc gamma R-mediated phagocytosis, with differentially expressed genes in nuclei of proliferation list cells vs. other cells.** One of the representative KEGG pathways, Fc gamma R-mediated phagocytosis, is visualized. Color shows cell typic difference in gene expression between sinus node myocytes and other cells. Red and green indicate genes expressed higher and lower in nuclei of proliferation list cells compared to other cells respectively. **Supplementary Figure 15. Visualization of the KEGG pathway, B cell receptor signaling pathway, with differentially expressed genes in nuclei of proliferation list cells vs. other cells.** One of the representative KEGG pathways, B cell receptor signaling pathway, is visualized. Color shows cell typic difference in gene expression between sinus node myocytes and other cells. Red and green indicate genes expressed higher and lower in nuclei of proliferation list cells compared to other cells respectively. Supplementary_Figures

Supplementary_Table1 **Excluded features.** Genes excluded because they didn’t pass the filter for high quality and highly expressed genes. Supplementary_Table1

Supplementary_Table2 **Gene lists.** Genes in Ed_list and related to proliferation are in Sheet 1 and Sheet 2 respectively. Supplementary_Table2

Supplementary_Table3 **AUCell analysis.** Each cell was assigned as proliferation related or not, or Ed_list genes related or not. Supplementary_Table3

Supplementary_Table4 **Analyses and results about sinus node myocytes.** Sheet1. DEG_ANOVA: differentially expressed genes (DEGs) in sinus node myocytes vs. others calculated with the method ANOVA. Sheet2. DEG_Hurdle: DEGs in sinus node myocytes vs. others calculated with the method Hurdle. Sheet3. Intersection: intersection of DEGs from Sheet 1 and Sheet 2. Sheet 4. GO: Gene Ontology (GO) enrichment in sinus node myocytes vs. others. Sheet 5. KEGG: Kyoto Encyclopedia of Genes and Genomes (KEGG) enrichment in sinus node myocytes vs. others. Supplementary_Table4

Supplementary_Table5 **Analyses and results about cells highly expressing genes in Ed list.** Sheet1. DEG_ANOVA: DEGs in cells highly expressing genes in Ed list vs. others calculated with the method ANOVA. Sheet2. DEG_Hurdle: DEGs in cells highly expressing genes in Ed list vs. others calculated with the method Hurdle. Sheet3. Intersection: intersection of DEGs from Sheet 1 and Sheet 2. Sheet 4. GO: GO enrichment in cells highly expressing genes in Ed list vs. others. Sheet 5. KEGG: KEGG enrichment in cells highly expressing genes in Ed list vs. others. Supplementary_Table5

Supplementary_Table6 **Analyses and results about cells highly expressing genes in proliferation list.** Sheet1. DEG_ANOVA: DEGs in cells highly expressing genes in proliferation list vs. others calculated with the method ANOVA. Sheet2. DEG_Hurdle: DEGs in cells highly expressing genes in proliferation list vs. others calculated with the method Hurdle. Sheet3. Intersection: intersection of DEGs from Sheet 1 and Sheet 2. Sheet 4. GO: GO enrichment in cells highly expressing genes in proliferation list vs. others. Sheet 5. KEGG: KEGG enrichment in cells highly expressing genes in proliferation list vs. others. Supplementary_Table6

Supplementary_Table7 **Comparison of enrichments.** Enrichments from sinus nodes cells, cells highly expressing genes in Ed list, and cells highly expressing genes in proliferation list are summarized and compared in this table. Supplementary_Table7

Supplementary_Table8 **Analysis methods.**Summary of each analysis and its reference. Supplementary_Table8

Supplementary_Table9 **Abbreviation.** Summary of abbreviations in this paper and their full names. Supplementary_Table9
