# Supplementary material for: Characterization of diverse populations of sinoatrial node cells and their proliferation potential at single nucleus resolution
Source: Heliyon. 2022 Dec 29;9(1):e12708. doi: 10.1016/j.heliyon.2022.e12708 (PMC9826826; doi:10.1016/j.heliyon.2022.e12708)

**Supplementary figures**

Supplementary Figure 1. Quality control.


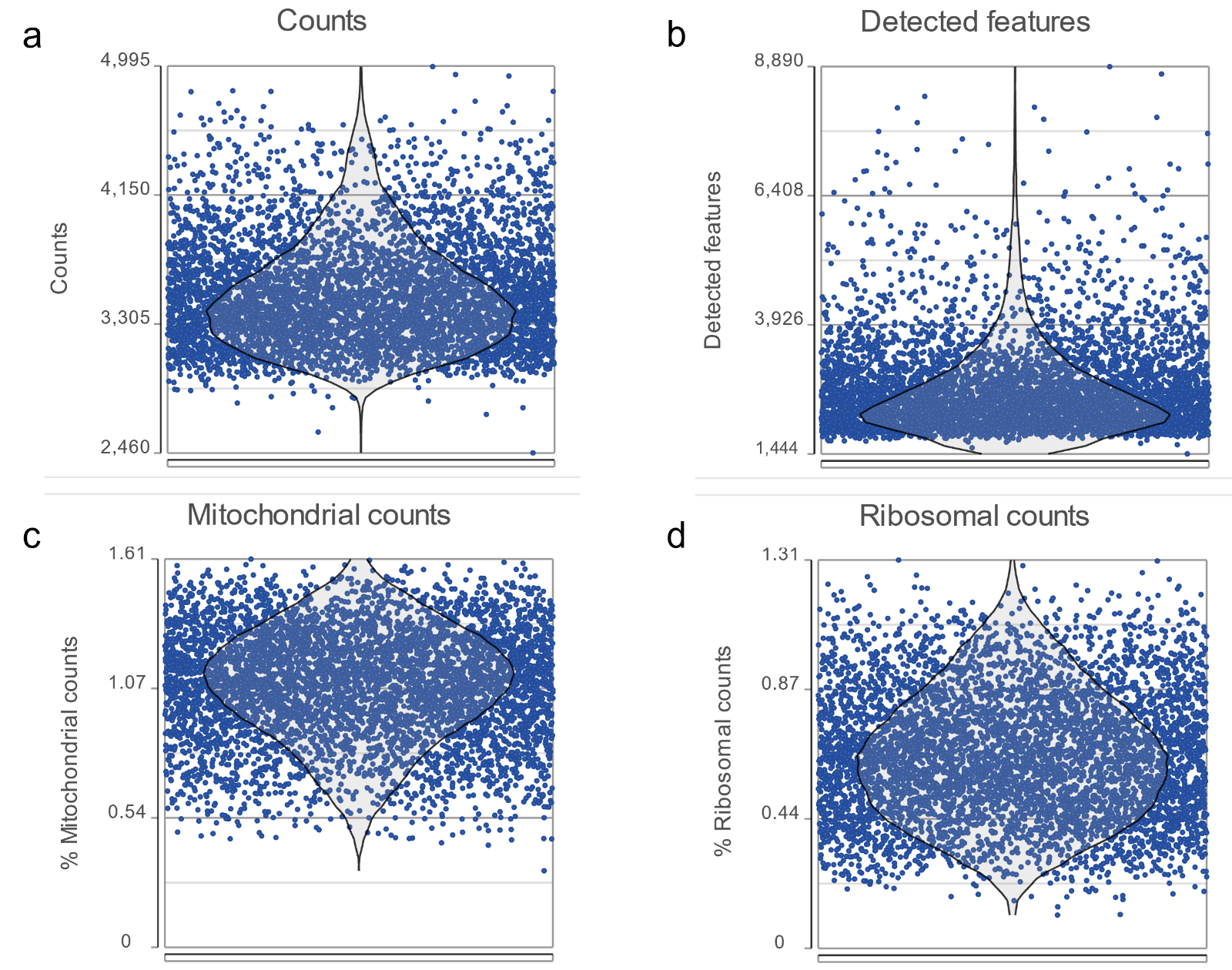


Supplementary Figure 2. Cell type proportion.


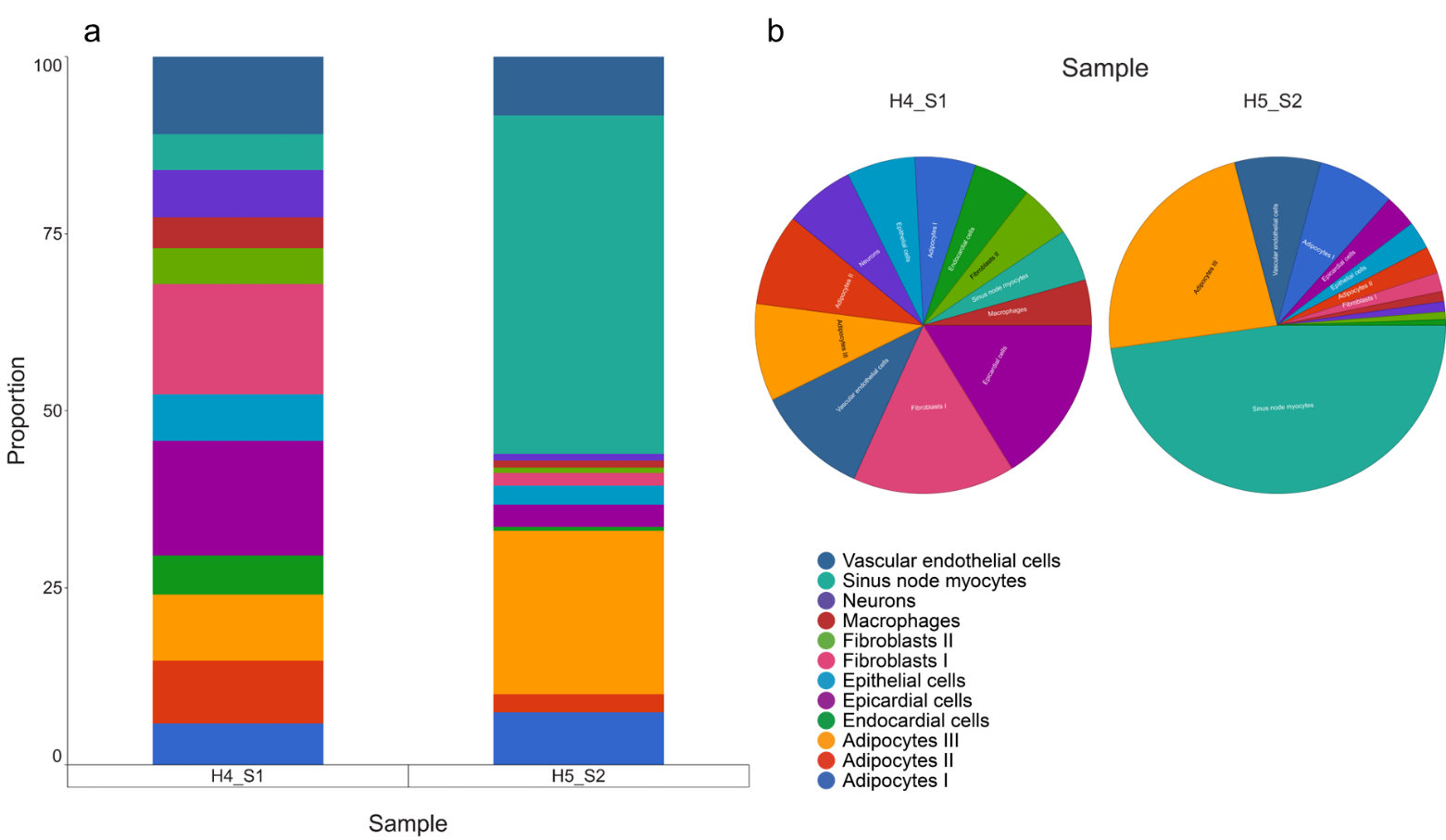


Supplementary Figure 3. Dimension reduction in PCA.


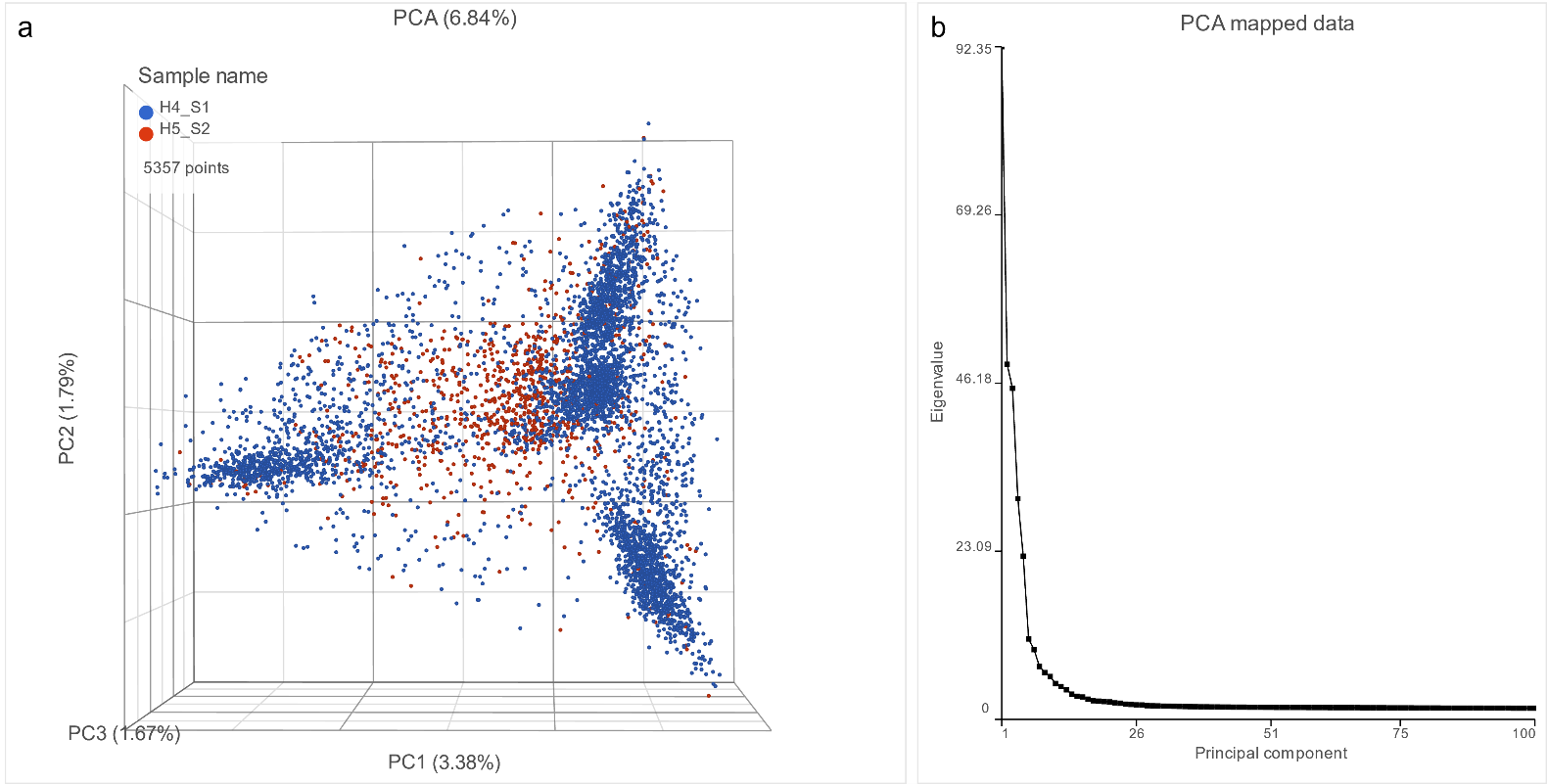


Supplementary Figure 4. Selection of best K parameter for K-means clustering.


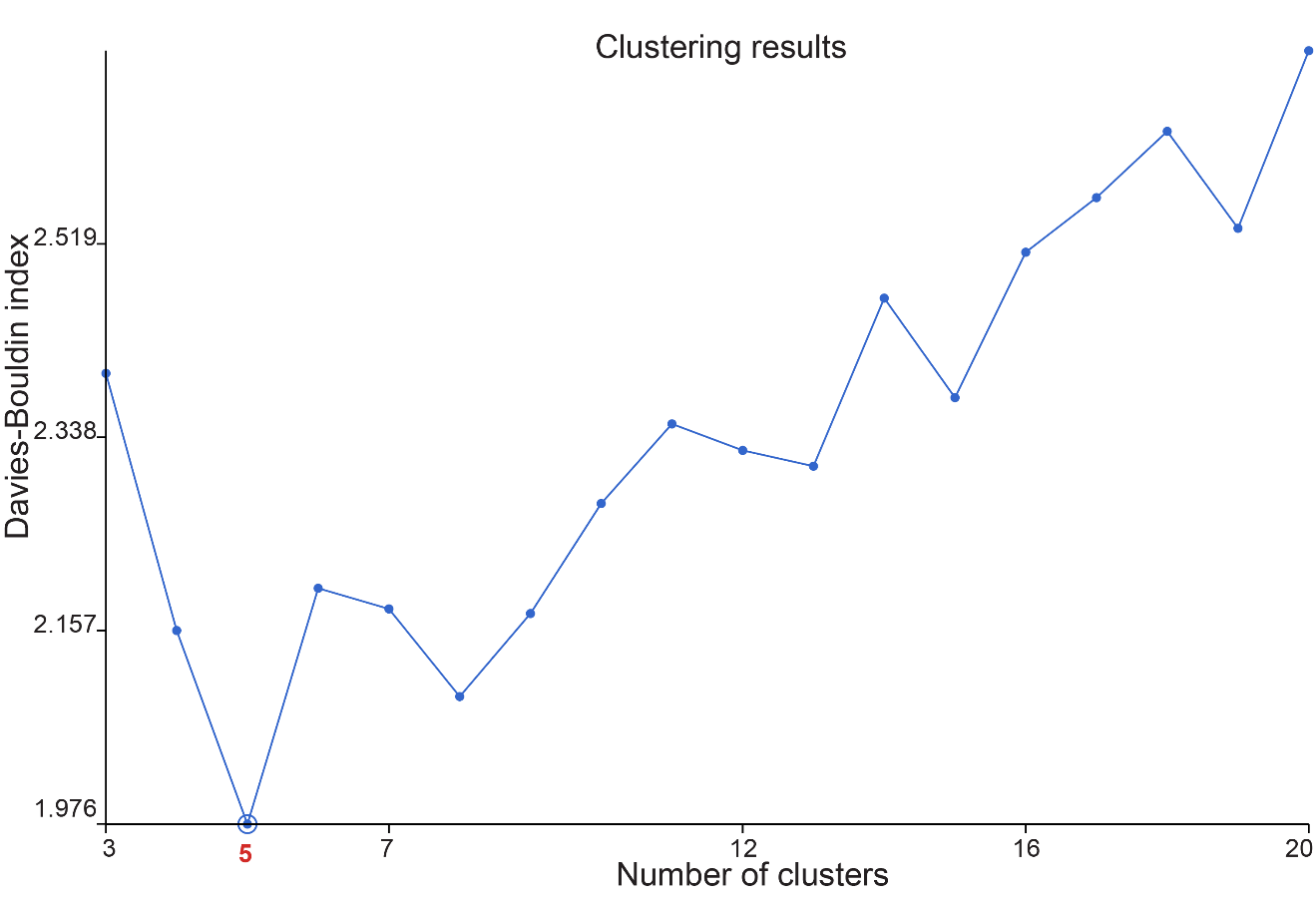


Supplementary Figure 5. T-distributed stochastic neighbor embedding (t-SNE) analysis.


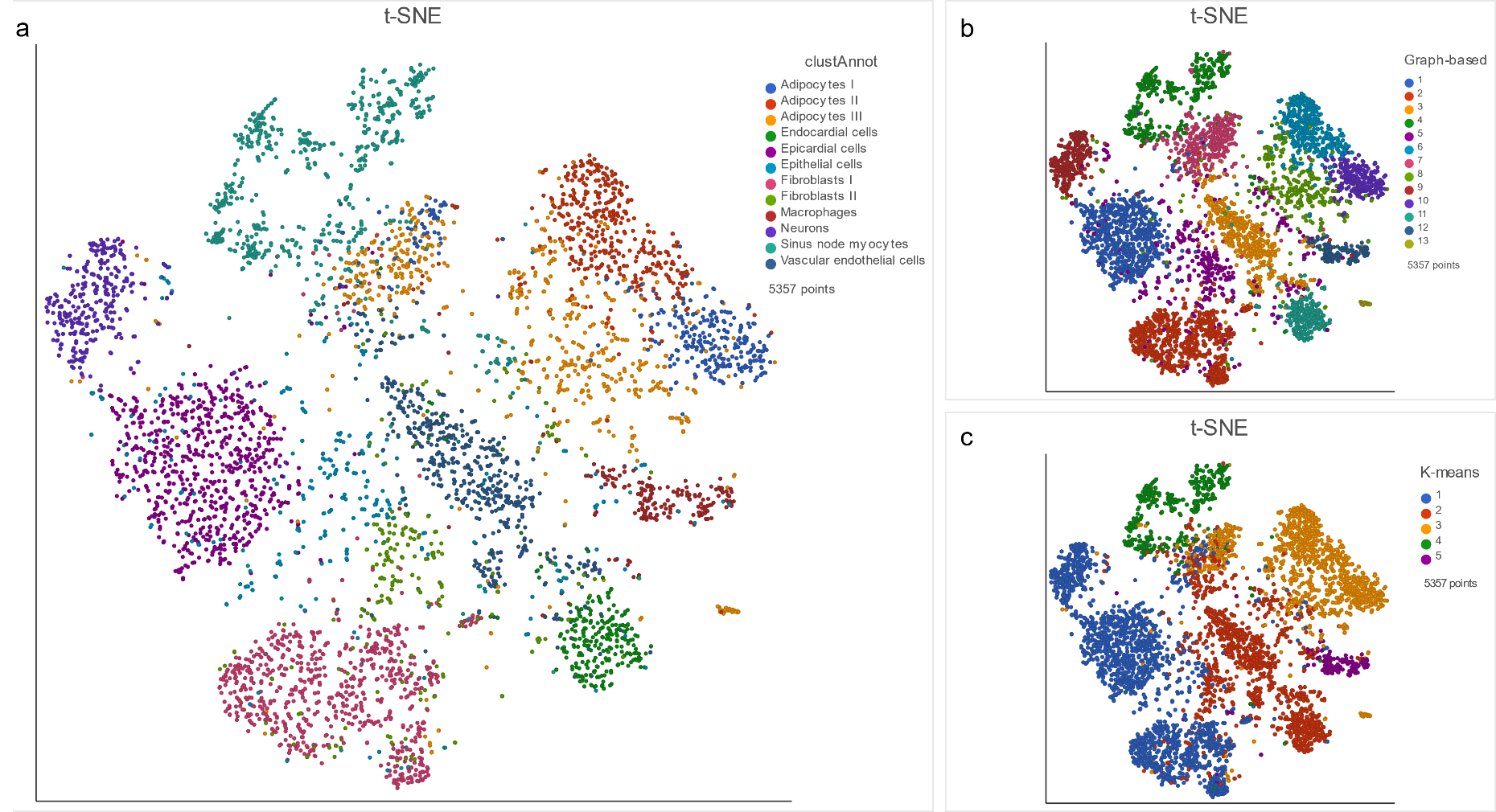


Supplementary Figure 6. Trajectory colored by clusters produced in graph-based and k-means clustering.


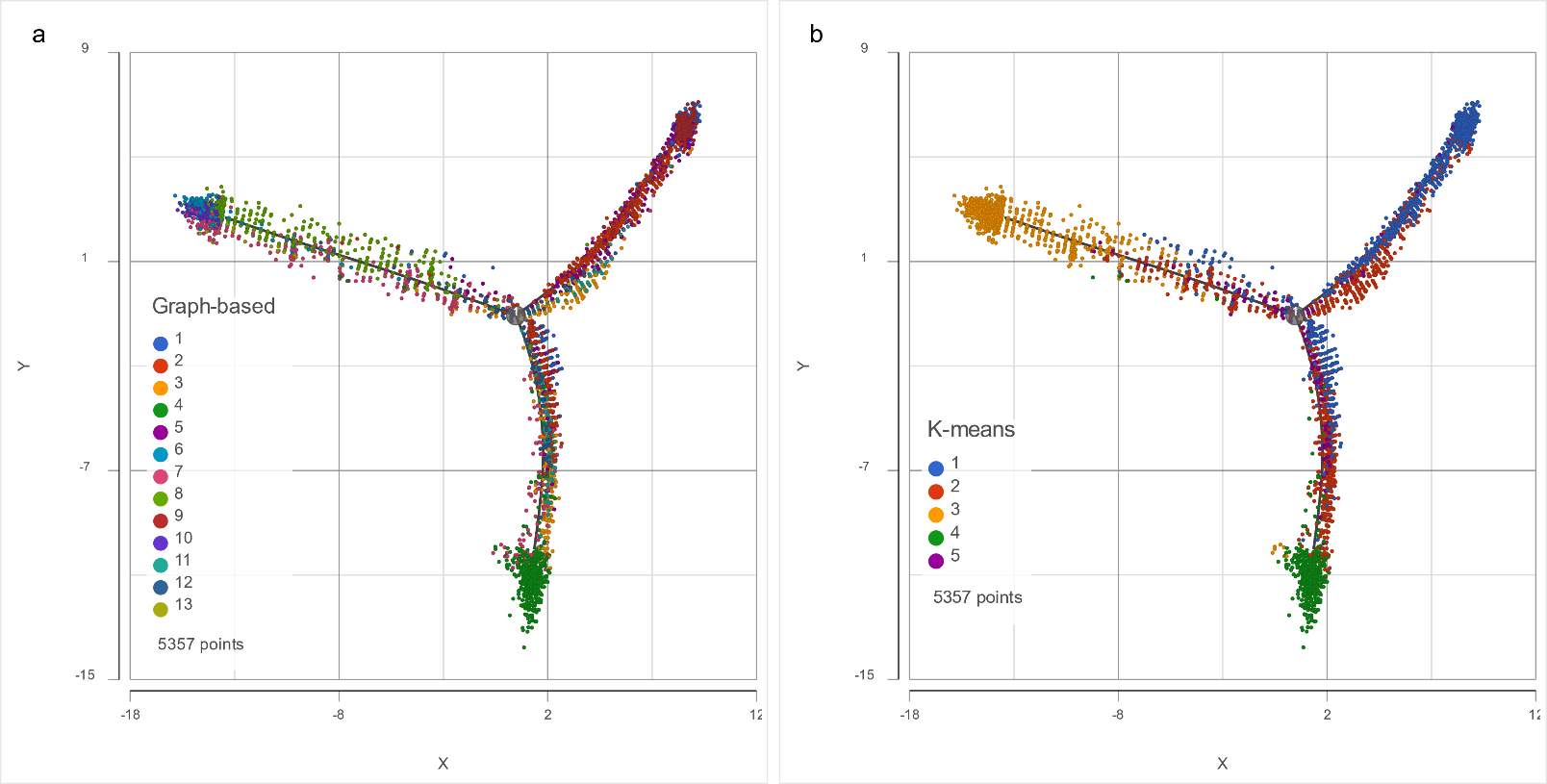


Supplementary Figure 7. Expression of Ed list genes in nuclei of all types of SAN cells.


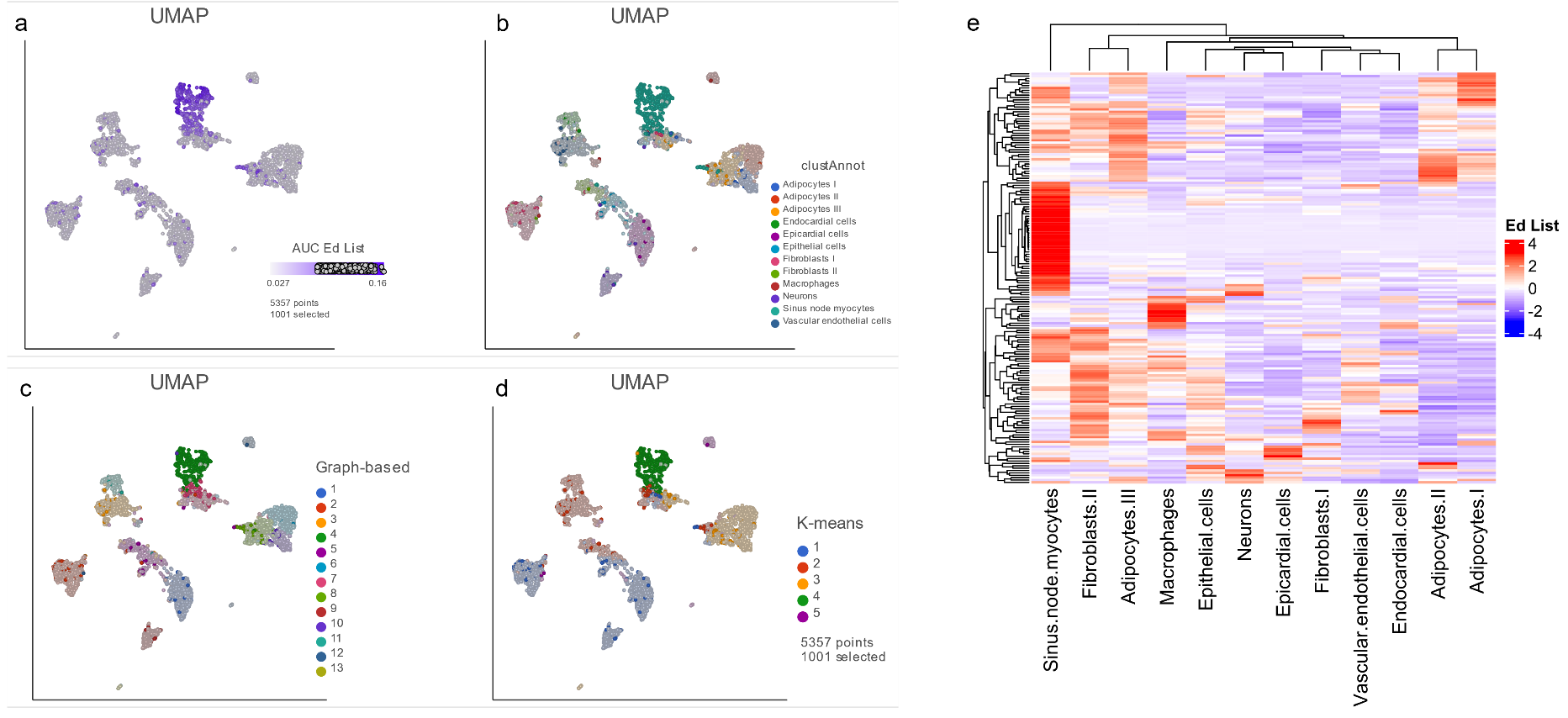


Supplementary Figure 8. Differentially expressed genes in nuclei of Ed list cells vs. other cells.


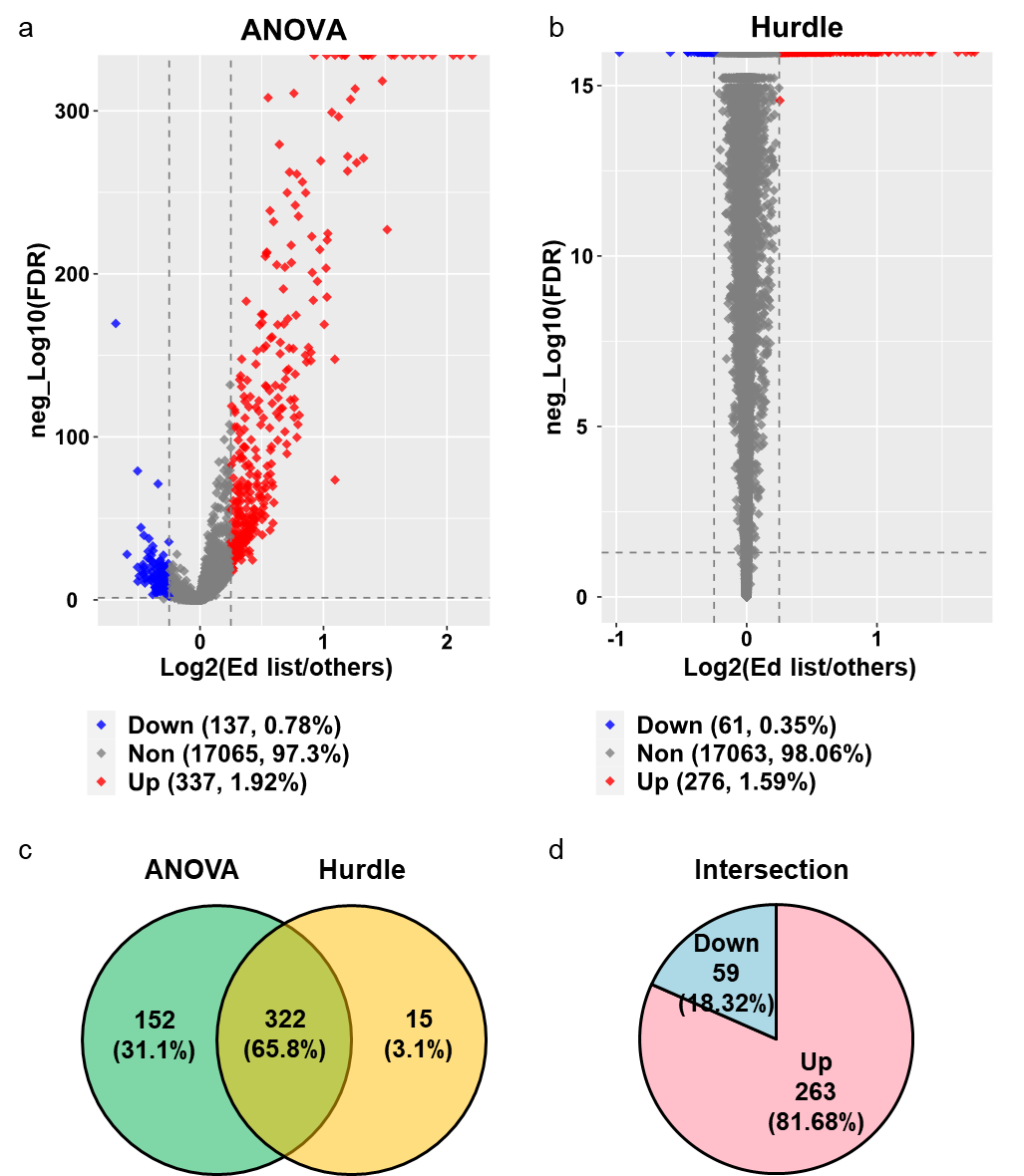


Supplementary Figure 9. Differentially enriched GO terms and KEGG pathways in nuclei of Ed list cells vs. other cells.


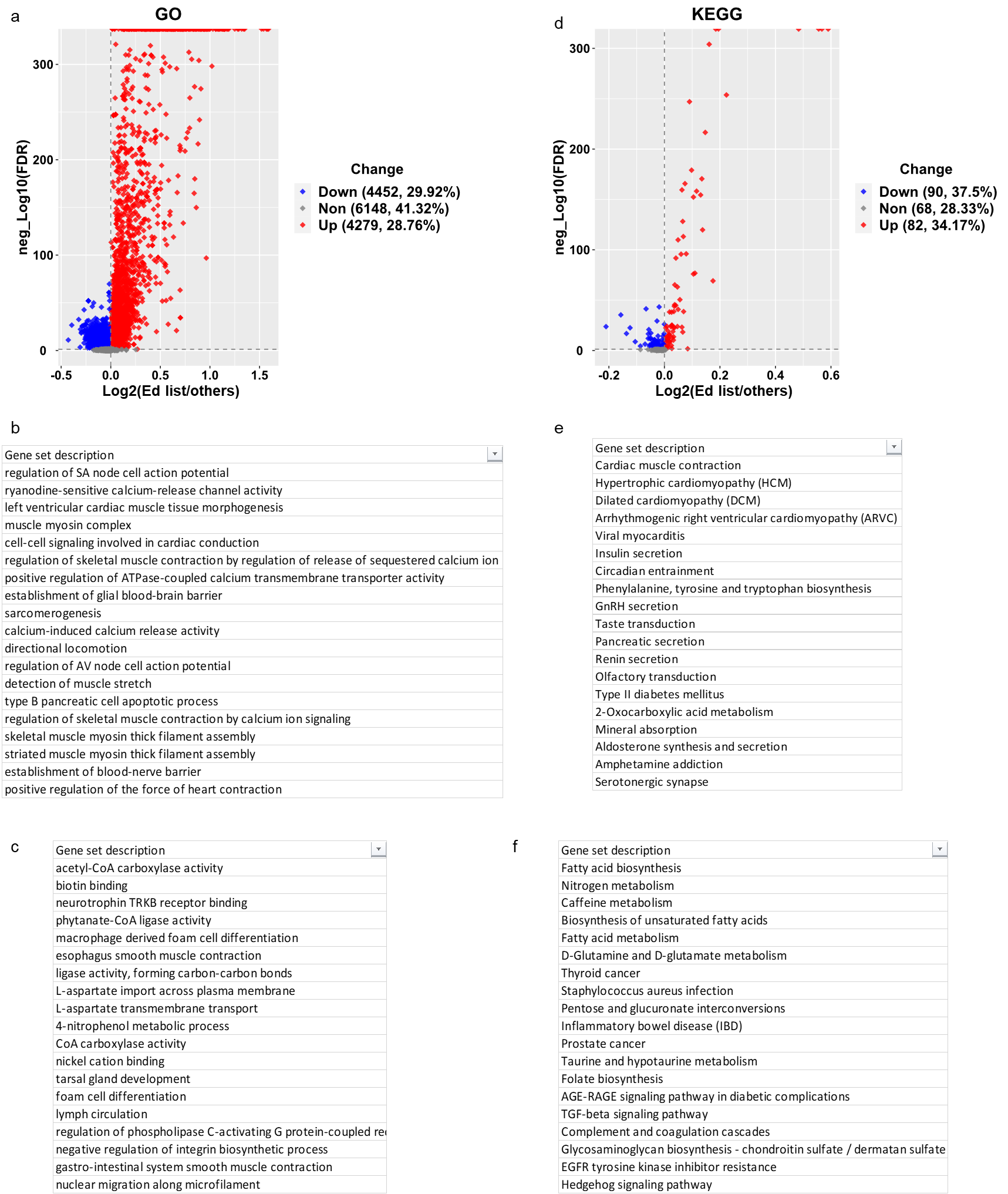


Supplementary Figure 10. Visualization of the KEGG pathway, fatty acid metabolism, with differentially expressed genes in nuclei of proliferation list cells vs. other cells.


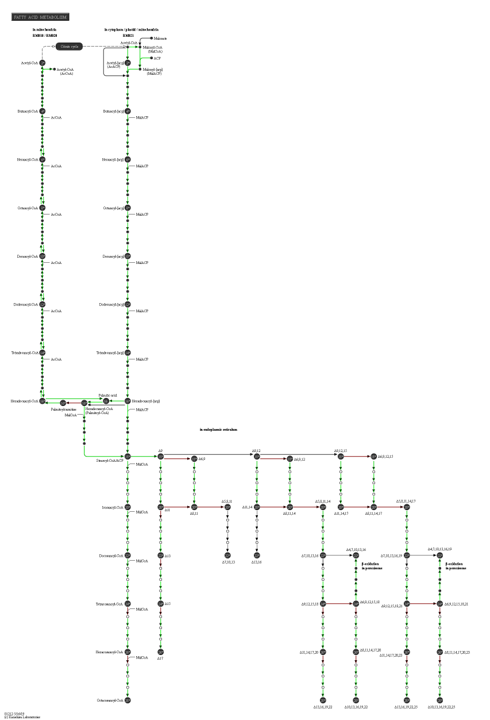


Supplementary Figure 11. Visualization of the KEGG pathway, propanoate metabolism, with differentially expressed genes in nuclei of proliferation list cells vs. other cells.


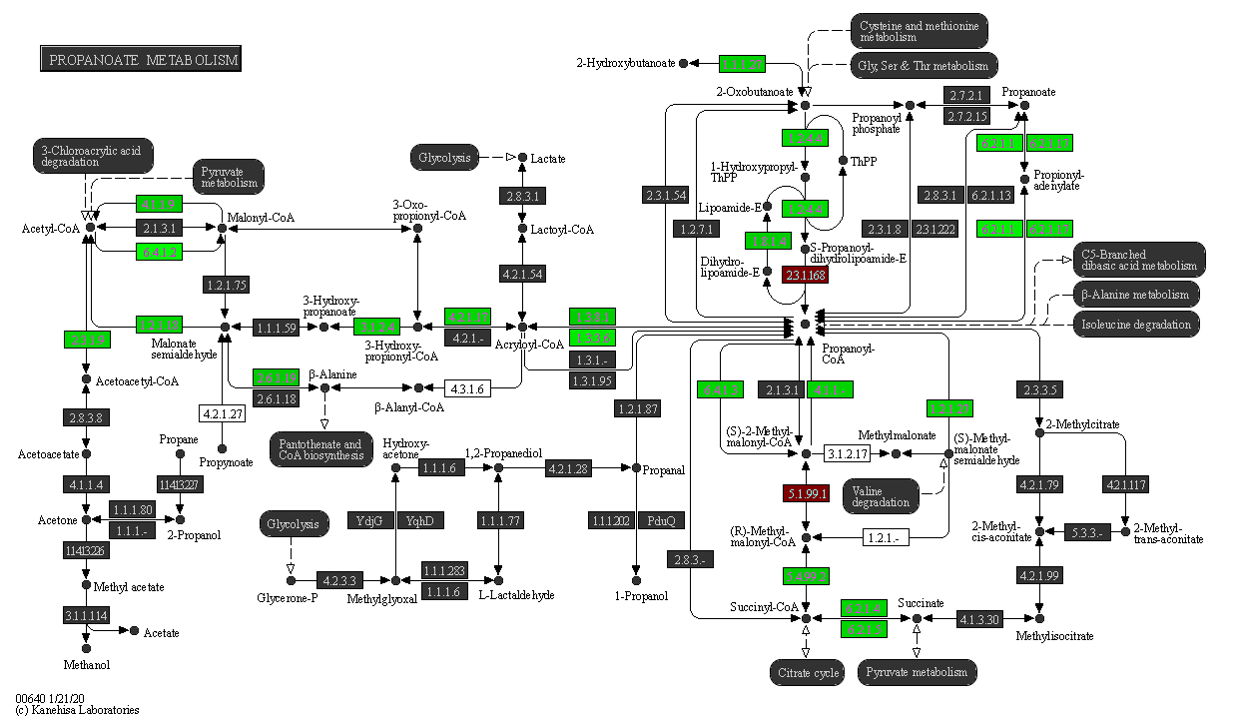


Supplementary Figure 12. Visualization of the KEGG pathway, PPAR signaling pathway, with differentially expressed genes in nuclei of proliferation list cells vs. other cells.


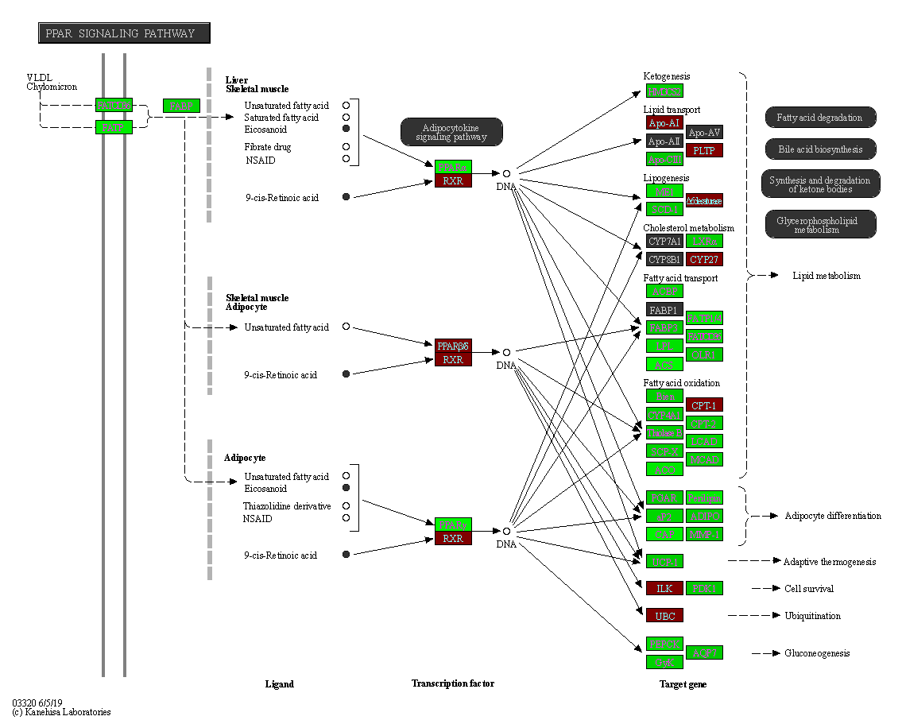


Supplementary Figure 13. Visualization of the KEGG pathway, p53 signaling pathway, with differentially expressed genes in nuclei of proliferation list cells vs. other cells.


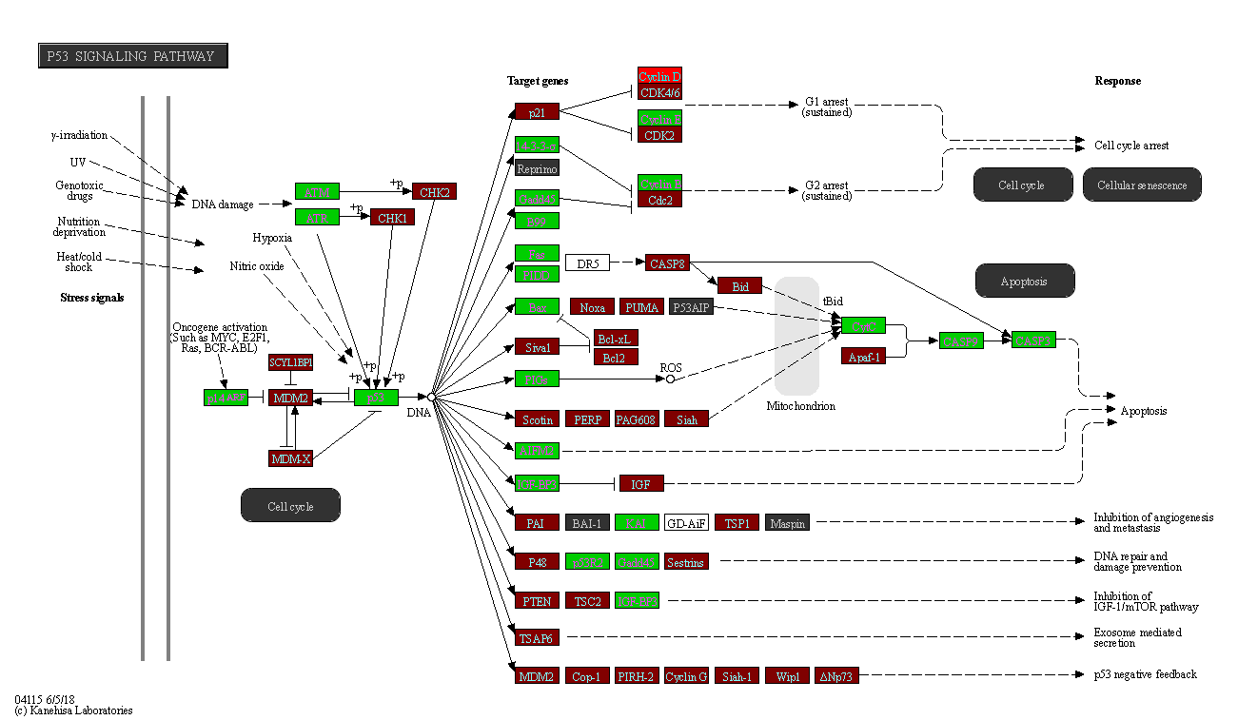


Supplementary Figure 14. Visualization of the KEGG pathway, Fc gamma R-mediated phagocytosis, with differentially expressed genes in nuclei of proliferation list cells vs. other cells.


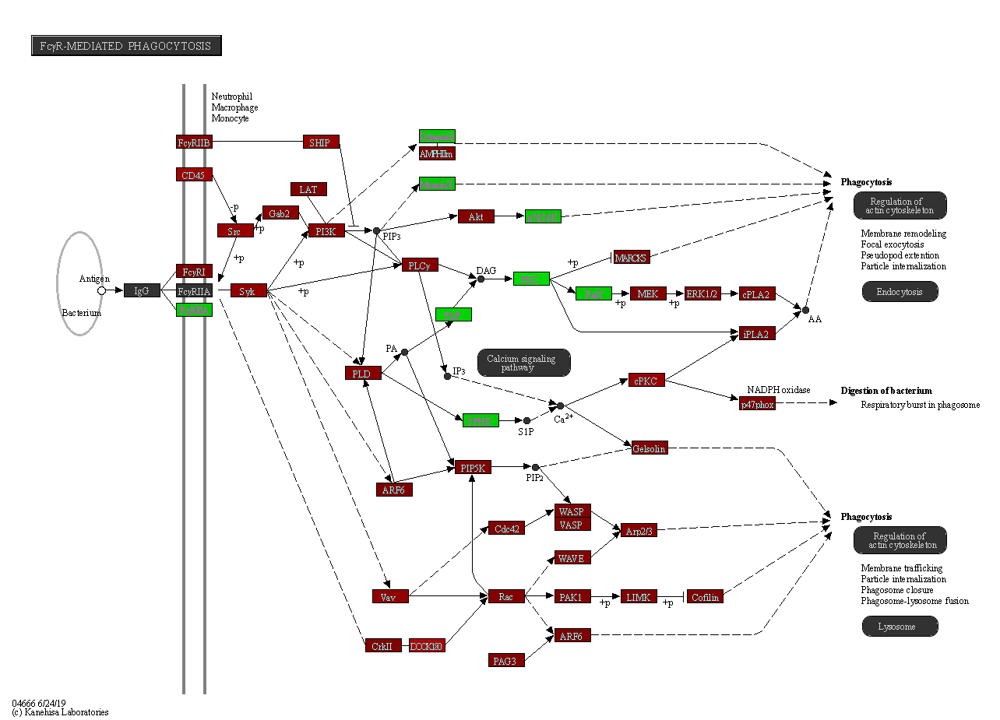


Supplementary Figure 15. Visualization of the KEGG pathway, B cell receptor signaling pathway, with differentially expressed genes in nuclei of proliferation list cells vs. other cells.


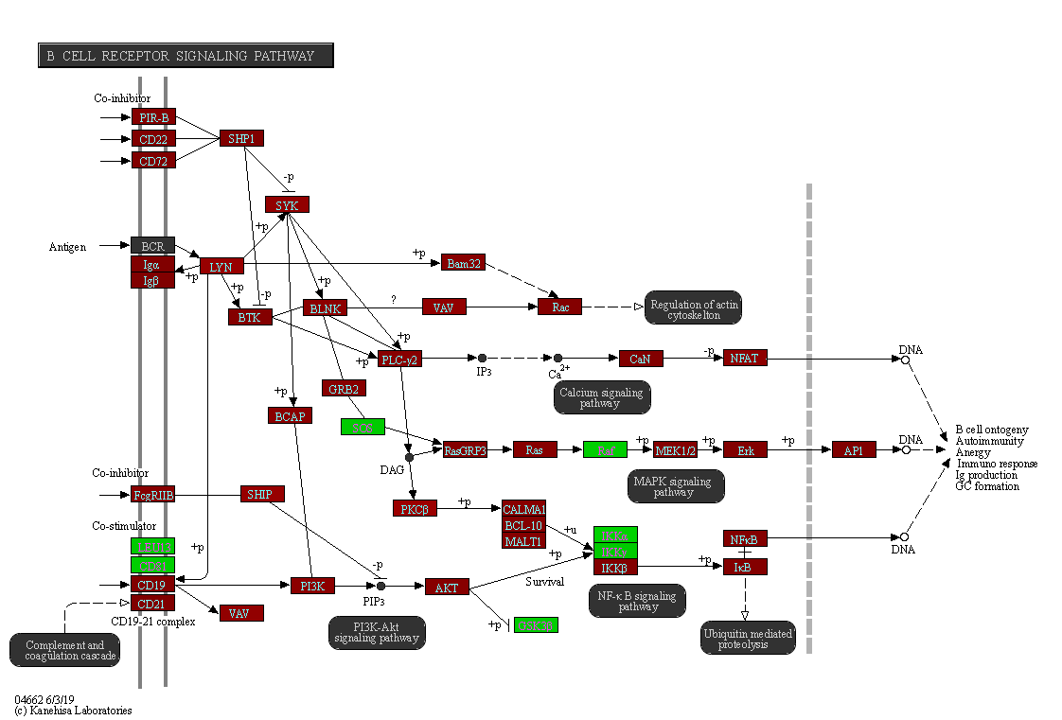

Supplement: Supplementary_Figures — Supplementary Figure 1. Quality control. Gene counts (a), expressed features (b), percentage of mitochondrial counts (c), and percentage of ribosomal counts (d) were displayed in scatter and violin plots. Supplementary Figure 2. Cell type proportion. Proportion of different types of cells that were annotated in the reference paper were displayed in stack bar plot (a) and pie plot (b). Supplementary Figure 3. Dimension reduction in PCA. Three-dimensional PCA plot of single nuclei in the two samples were displayed in (a), and their eigen values were shown in scree plot (b). x-axis represents the first principal component, y-axis represents the second principal component, and z-axis represents the third principal component. Nuclei from two samples were in two different colors, blue or red. Supplementary Figure 4. Selection of best K parameter for K-means clustering. The value, k=5, corresponding to the smallest Davies-Bouldin index is the best k parameter. Supplementary Figure 5. T-distributed stochastic neighbor embedding (t-SNE) analysis. The single nuclei were displayed in t-SNE with the first 20 principal components and colored by cluster annotation in the reference paper(a), graph-based cluster in this study (b), and K-means cluster (k = 5) in this study (c). Supplementary Figure 6. Trajectory colored by clusters produced in graph-based and k-means clustering. The single nuclei were also colored by graph-based cluster in this study (a) and K-means cluster (k = 5) in this study (b). Supplementary Figure 7. Expression of Ed list genes in nuclei of all types of SAN cells. Top nuclei expressing gene in the Ed list are displayed in feature plots in the projection of UMAP and colored by AUCell score (a), cluster annotation in the reference paper (b), graph-based cluster in this study (c), and K-means cluster (k = 5) in this study (d). E. Heatmap of expression of Ed list genes and hierarchy clustering of cell types and genes. Supplementary Figure 8. Differentially expres [file mmc1.docx]
